# Untargeted metabolomics reveals serum metabolites related to energy metabolism and inflammation associated with juvenile dermatomyositis

**DOI:** 10.1007/s11306-026-02425-5

**Published:** 2026-04-29

**Authors:** Kaylie I. Kirkwood-Donelson, Dylan J. Johnson, Payam Noroozi Farhadi, Kakali Sarkar, Adam I. Schiffenbauer, Frederick W. Miller, Gregory Kudzin, Erin S. Baker, Jian-Liang Li, Lisa G. Rider, Alan K. Jarmusch

**Affiliations:** 1 Mass Spectrometry Research Center, Immunity, Inflammation and Disease Laboratory, National Institute of Environmental Health Sciences, National Institutes of Health, Durham, NC USA; 2 Integrative Bioinformatics Support Group, Biostatistics and Computational Biology Branch, National Institute of Environmental Health Sciences, National Institutes of Health, Durham, NC USA; 3 Environmental Autoimmunity Group, Clinical Research Branch, National Institute of Environmental Health Sciences, National Institutes of Health, Bethesda, MD USA; 4 Department of Chemistry, University of North Carolina at Chapel Hill, Chapel Hill, NC USA

**Keywords:** Juvenile dermatomyositis, Untargeted metabolomics, Serum metabolomics, Inflammation, Energy metabolism

## Abstract

**Introduction:**

Juvenile dermatomyositis (JDM) is a rare autoimmune inflammatory myopathy characterized by muscle weakness and distinctive skin rashes. Despite advancements in the clinical understanding of JDM, metabolic disturbances underlying the disease remain poorly understood.

**Objectives:**

This study aimed to investigate serum metabolite differences in JDM compared to age- and sex-matched unaffected siblings (US) and unrelated healthy controls (HC), and to identify metabolite abundance differences associated with disease severity.

**Methods:**

Serum samples from JDM (n = 16) and adult dermatomyositis (DM; n = 15) patients and corresponding US and HC underwent untargeted metabolomics profiling. Multivariate, univariate, and correlation analyses were employed to identify metabolites differentiating groups and correlating with Physician Global Damage (PGD) scores.

**Results:**

JDM patients exhibited modest but discernible alterations in serum metabolites compared to controls, many of which also correlated with PGD. Several bioactive lipids and pyroglutamic acid were upregulated in JDM and positively correlated with PGD. Changes in xanthine, methionine and N-acetylneuraminic acid also indicated increased oxidative stress and inflammation. Markers of increased energy demand and muscle damage, including acylcarnitines, creatine, 4-guanidinobutyric acid, glutamine, and phenylacetylglutamine, were differential and correlated with PGD in some cases. A metabolite abundance gradient from JDM to US to HC groups suggests that siblings help account for genetic and environmental influences on the metabolome. DM patients did not show significant serum changes compared to US.

**Conclusion:**

Untargeted metabolomics revealed distinct serum metabolite alterations in JDM, providing insights into disease-related metabolic perturbations. These findings enhance understanding of JDM pathophysiology and inform future large-scale, targeted studies.

**Supplementary Information:**

The online version contains supplementary material available at 10.1007/s11306-026-02425-5.

## Introduction

Dermatomyositis (DM) is a systemic autoimmune disorder characterized by muscle inflammation, weakness, and characteristic skin rashes. DM is rare in adults and juveniles (JDM); however, it is the most common idiopathic inflammatory myopathy (IIM) in children (McCann et al., 2006; Shah et al., 2013). Diagnosis is based on a combination of clinical features (e.g., muscle weakness and distinct rashes), laboratory results such as elevated serum creatine kinase (CK) or aldolase, muscle biopsy evidence of myositis, and supportive testing including myositis-specific autoantibodies (Bohan & Peter, 1975a; Lundberg et al., 2017). Diagnosis and disease monitoring can be complicated by disease heterogeneity and shared characteristics between several systemic autoimmune rheumatic diseases (SARDs). The pathogenesis of DM is not fully elucidated but involves environmental and immunological factors in genetically susceptible individuals (Chen et al., 2024; Lin et al., 2023).

Several studies have begun investigating the metabolomic profiles of DM and other IIMs in plasma, serum and urine. These studies revealed alterations in metabolites related to amino acid biosynthesis, fatty acids and beta-oxidation, bile acids, and purine metabolism. For example, Zhang et al. identified several amino acids (glutamine, methionine, isoleucine, tryptophan, glutamic acid, and phenylalanine) as potential biomarkers in DM plasma compared to healthy controls (Zhang et al., 2019). Results from later studies expanded on these findings with comparison to other IIMs including polymyositis (PM), anti-synthetase syndrome and immune-mediated necrotizing myopathy, as well as evaluation of myositis-specific autoantibody-defined subtypes. Zhao et al. identified panels of five to seven metabolites that differentiated three IIMs from each other and from healthy controls (Zhao et al., 2023). Others observed alterations in lipids such as fatty acids, bile acids, and acylcarnitines that distinguished myositis-specific autoantibody DM subgroups (Liu et al., 2022; Wang et al., 2024). These studies focused on adult populations; information on metabolism in JDM is limited. One longitudinal targeted study of serum amino acids, acylcarnitines, and sphingolipids reported that long-chain acylcarnitines and ceramides were associated with JDM prior to treatment (Dvergsten et al., 2022). This work was limited by the analytes targeted and small sample size. Thus, further investigation is needed to reveal comprehensive metabolomic changes associated with JDM.

In this work, we utilized untargeted metabolomics to investigate serum metabolite differences in DM and JDM patients compared to their sex-matched, close-in-age unaffected siblings (US) and to unrelated healthy controls (HC). The use of US is particularly valuable to control for some genetic and environmental factors when pursuing alterations with high specificity. We also sought to identify metabolites correlated with physician global damage (PGD) scores, which capture cumulative disease burden, physical deconditioning, and sustained inflammation and treatment exposure. Through this untargeted metabolomic profiling approach, we have built upon previous findings by identifying metabolic differences that distinguish disease status and associate with cumulative disease burden. These results highlight candidate metabolites and biological processes for follow-up mechanistic and targeted investigations.

## Methods

### Study population and sample collection

JDM and DM patients their close-in-age, same sex unaffected siblings, and demographically matched healthy controls without autoimmune disease and living in the United States or Canada were enrolled through the National Institutes of Health Clinical Center in the National Institute of Environmental Health Sciences Twin-Sibling Study of Systemic Rheumatic Diseases (NCT00055055) between 2003 to 2021. Probands were within 5 years of diagnosis at enrollment and met probable or definite criteria for JDM/DM (Bohan and Peter, NEJM, 1975). A same-sex sibling within 5 years of the proband’s age and without any autoimmune disease were also enrolled, as well as same sex healthy controls within 5 years of JDM and 10 years of adult DM patients. Control subjects were unrelated to probands and had no history of autoimmune disease. All participants underwent a standardized medical assessment and provided blood samples. Patient demographic information and relevant clinical features are summarized in Table 1. Patient medications are listed in Supplementary Table S1. The study was approved by the NIH Institutional Review Board; written informed consent was obtained from all participants. Patients with anti-Jo-1 autoantibodies (1 JDM, 1 DM) were excluded from analysis as those with anti-aminoacyl tRNA synthetase autoantibodies are considered a distinct subgroup of DM (Faghihi-Kashani et al., 2025). The remaining autoantibody-positive counts include 6/16 anti-TIF1γ, 4/16 anti-NXP2, and 2/16 anti-MDA5 JDM patients; 4/15 anti-TIF1γ, and 1/15 anti-NXP2 DM patients.

**Table 1:** Demographic and Clinical Features for Samples Included in Metabolomic Analysis

Group	Juvenile Dermatomyositis			Adult Dermatomyositis		
Class	Healthy controls (n = 16)	Unaffected Siblings (n = 16)	Juvenile DM (n = 16)	Healthy controls (n = 15)	Unaffected Siblings (n = 15)	Adult DM (n = 15)
Visit Age	14.47 (3.59) [16]	15.72 (3.75) [16]	15.58 (1.88) [16]	45.36 (17.19) [15]	47.83 (15.34) [15]	47.99 (15.99) [15]
Sex	FEMALE 8; MALE 8 [16]	FEMALE 8; MALE 8 [16]	FEMALE 8; MALE 8 [16]	FEMALE 13; MALE 2 [15]	FEMALE 13; MALE 2 [15]	FEMALE 13; MALE 2 [15]
Race	ASIAN 1; BLACK OR AFRICAN AMERICAN 1; WHITE 14 [16]	ASIAN 1; BLACK OR AFRICAN AMERICAN 1; WHITE 14 [16]	ASIAN 1; BLACK OR AFRICAN AMERICAN 1; WHITE 14 [16]	BLACK OR AFRICAN AMERICAN 4; WHITE 11 [15]	BLACK OR AFRICAN AMERICAN 2; UNKNOWN 1; WHITE 12 [15]	BLACK OR AFRICAN AMERICAN 2; WHITE 13 [15]
Ethnicity	HISPANIC OR LATINO 1; NOT HISPANIC OR LATINO 15 [16]	HISPANIC OR LATINO 3; NOT HISPANIC OR LATINO 13 [16]	HISPANIC OR LATINO 2; NOT HISPANIC OR LATINO 14 [16]	NOT HISPANIC OR LATINO 15 [15]	NOT HISPANIC OR LATINO 13; UNKNOWN/NOT REPORTED 2 [15]	NOT HISPANIC OR LATINO 15 [15]
Diagnosis Age or Reference Age	NA	NA	14.06 (2) [16]	NA	NA	46.84 (16.37) [15]
Disease Duration (months)	NA	NA	17.81 (14.2) [16]	NA	NA	13.33 (10.66) [15]
Physician Global Disease Activity (PGA, 0–100 mm)	NA	NA	18 (18.78) [16]	NA	NA	21.53 (23.88) [15]
Physician Global Disease Damage (PGD, 0–100 mm)	NA	NA	8.31 (11.29) [16]	NA	NA	18.93 (28.49) [15]
HAQ/CHAQ Disability Index	NA	NA	0.55 (0.81) [16]	NA	NA	0.63 (0.76) [15]
VAS Physician Global Damage	NA	NA	0.94 (0.74) [8]	NA	NA	0.85 (1.2) [6]
ALP	135 (77.13) [13]	123.94 (105.57) [8]	109.14 (78.51) [7]	68.74 (25.89) [11]	68.67 (7.79) [6]	48.72 (18.72) [9]
ALT	19.31 (7.2) [13]	22.12 (8.32) [8]	28.79 (11.47) [7]	18.4 (12) [12]	28.83 (17.3) [6]	38.39 (14.01) [9]
AST	21.92 (4.65) [13]	22.5 (11.59) [8]	24.79 (8.4) [7]	18.75 (3.54) [11]	23.5 (9.61) [6]	33.33 (26.55) [9]
BILIRUBIN Direct	0.12 (0.04) [10]	0.17 (0.1) [4]	0.17 (0.06) [3]	0.11 (0.02) [5]	0.1 (0) [3]	0.14 (0.05) [5]
BILIRUBIN Total	0.65 (0.3) [13]	0.59 (0.45) [8]	0.49 (0.24) [7]	0.59 (0.21) [11]	0.57 (0.28) [6]	0.53 (0.25) [9]
BMI	20.18 (2.61) [14]	21.43 (2.65) [10]	22.73 (4.02) [12]	27.06 (4.55) [14]	25.41 (6.05) [12]	26.94 (8.15) [12]
CHOLESTEROL Total	NA	191 (NA) [1]	161.67 (29.28) [6]	171.75 (23.99) [8]	146 (NA) [1]	169.29 (58.93) [7]
CK Total (U/L)	125.73 (67.78) [15]	190.5 (290.94) [12]	127.42 (90.39) [12]	173.57 (283.65) [14]	114.71 (41.76) [7]	263.67 (370.2) [12]
Uric Acid (mg/dL)	4.69 (1.6) [13]	4.74 (1.24) [7]	4.8 (0.97) [6]	4.44 (1.25) [14]	4.87 (1.45) [7]	4.56 (1.39) [11]
GGT	13 (NA) [1]	NA	31.12 (23.43) [4]	53 (NA) [1]	NA	NA
HDL	NA	67 (NA) [1]	54.33 (22.99) [6]	63.88 (16.43) [8]	47 (NA) [1]	72 (18.82) [7]
HEIGHT (cm)	160.09 (16.47) [14]	162.66 (14.42) [10]	167.67 (9.49) [12]	166.48 (7.77) [14]	164.62 (5.39) [12]	166.44 (5.35) [12]
LDL	NA	98 (NA) [1]	81.6 (21.35) [5]	86.12 (14.67) [8]	83 (NA) [1]	80.57 (52.64) [7]
TRIGLYCERIDES	NA	133 (NA) [1]	156.5 (151.31) [6]	109.75 (60.94) [8]	80 (NA) [1]	84 (33.99) [7]
Consent Date (Year)	2007.56 (3.74) [16]	2013.31 (5.06) [16]	2013.31 (5.06) [16]	2011.2 (5.94) [15]	2009.73 (4.65) [15]	2009.67 (4.73) [15]
Date Drawn (Year)	2007.5 (3.67) [16]	2013.38 (5.15) [16]	2013.38 (5.15) [16]	2011.2 (5.94) [15]	2009.8 (4.71) [15]	2009.73 (4.65) [15]
Enrollment Date (Year)	2007.56 (3.74) [16]	2013.38 (5.15) [16]	2013.38 (5.15) [16]	2011.2 (5.94) [15]	2009.73 (4.65) [15]	2009.67 (4.73) [15]
Research Sample Date (Year)	2007.56 (3.74) [16]	2013.38 (5.15) [16]	2013.38 (5.15) [16]	2011.2 (5.94) [15]	2009.8 (4.71) [15]	2009.73 (4.65) [15]

Values given as mean (standard deviation) or n (%) [number of samples with data available]

### Sample preparation

Serum samples were prepared in batches with approximately the same percentage of sample group, sex distribution, and age range as the full study population. Trios of matched patient, US, and HC samples were prepared within the same batch to reduce extraction variability between sample trios. An aliquot of NIST Standard Reference Material (SRM) 909c human serum was prepared alongside each batch. Using a Hamilton liquid handler, 50 µL of each sample was transferred to a new microcentrifuge tube, then 200 µL of cold methanol was added. Samples were vortexed for 30 s then incubated at -20 °C for 30 min to facilitate protein precipitation. Extracts were centrifuged for 10 min at 14,000 rcf and 4 °C, then 150 µL of supernatant was manually transferred to new microcentrifuge tubes. Supernatants were dried using a GeneVac EZ-2 Plus on the HPLC program for one hour. Dried extracts were stored at -80 °C until analysis. Prior to analysis, sample extracts were resuspended in 100 µL of 98:2 water/acetonitrile with the Hamilton liquid handler, vortexed for 30 s, then centrifuged for 5 min at 14,000 rcf and 4 °C. The Hamilton liquid handler was also used to generate a pooled quality control (QC) sample using 10 µL of each sample, then the experimental samples, NIST SRM serum, QC sample, and blanks comprised of the same resuspension solvent were transferred to glass autosampler vials.

### Metabolomics analysis

Untargeted metabolomics data were acquired on a platform coupling a Vanquish Horizon ultra-high performance liquid chromatography system (UPLC, ThermoFisher Scientific) and an Orbitrap Fusion Tribrid high-resolution mass spectrometer (MS, ThermoFisher Scientific). Prior to analysis, the mass spectrometer was calibrated using FlexMix (ThermoFisher Scientific). EASY-IC (Thermo Scientific) fluoranthene was used during data collection as a lock mass. UPLC separation was carried out on a Kinetex F5 analytical column (2.1 mm inner diameter, 100 mm length, 100 Å, 2.6 µm particle size, Phenomenex) with an F5 guard cartridge at 30 °C. Gradient elution was performed using water with 0.1% acetic acid (mobile phase A) and acetonitrile with 0.1% acetic acid (mobile phase B). Separation was performed as follows: 0% B from 0—2.0 min, 0% to 100% B from 2.0 to 10.5 min, 100% B from 10.5 to 12.0 min, 100% to 0% B from 12.0 to 13.0 min, 0% B from 13.0 to 20.0 min at a flow rate of 0.5 mL/min. An EASY-Max NG ionization source was operated in the heated-electrospray ionization configuration. The source parameters were as follows: + 4000 V spray voltage in positive mode or -3000 V in negative mode, 50 arbitrary units (arb) sheath gas, 10 arb auxiliary gas, 1 arb sweep gas, ion transfer tube at 325 °C, and vaporizer at 350 °C.

MS data were collected in positive and negative ionization mode for all experimental samples, system blanks, pooled QC, and NIST SRM serum samples with 4 µL injection volumes. Batches were run separately, and the injection order within each batch was randomized. Data were collected for the pooled QC and NIST SRM serum samples at the beginning, middle, and end of each batch. System blanks were run every eight samples. The pooled QC was injected at different volumes (triplicate injections at 2 µL, 4 µL, and 6 µL) for signal response evaluation (Overdahl et al., 2023). MS data were acquired at 120,000 resolution from *m/z* 100–1000 with an RF lens of 60% and maximum injection time of 50 ms. Prior to acquiring sample data in positive or negative mode, MS/MS data were acquired using the AcquireX deep scan methodology with seven pooled QC MS/MS injections. MS/MS data were acquired at 30,000 resolution with an isolation width of *m/z* 1.5, stepped assisted higher-energy collision induced dissociation was used with energy steps of 20, 35, and 60 normalized collision energy, and a maximum injection time of 54 ms. An intensity filter was applied with an intensity threshold of 2 × 1e4. Dynamic exclusion was used with the following parameters: exclude after n = 3 times; if occurs within 15 s; exclusion duration of 6 s; a low mass tolerance of 5 ppm mass error; a high mass tolerance of 5 ppm mass error; and excluding isotopes.

### Data processing and statistical analyses

Data files (.raw) were processed with Compound Discoverer 3.3.3.220 (ThermoFisher Scientific) to identify and annotate molecular features. Features were assigned putative annotations based on MS/MS spectral matching in alignment with the Metabolomics Standards Initiative (Sumner et al., 2007). Public, commercial and in-house MS/MS spectral libraries including NIST2020, GNPS (accessed 04–01-2022), and mzCloud (offline, endogenous metabolites) were utilized. An output table containing feature descriptors, annotations, and peak areas was further processed in R using a Jupyter Notebook for data formatting and analysis. Initial processing steps included assigning MSI levels of confidence. To achieve MSI Level 1 identification, features were manually validated after being matched to an in-house library containing *m/z* and retention time data, acquired under identical conditions using the same analytical platform. MSI Level 2 was assigned to features having an MS/MS match to either a database entry or an in-house library entry. MSI Level 4 and Level 5 features have either an MS/MS match but no database match, or no MS/MS match. Feature quality was assessed via pooled QC signal response and dispersion ratio. The signal response evaluation approach utilized here is detailed in Overdahl et al. (Overdahl et al., 2023). Pearson correlation was used to assess the relationship between signal intensity and the three pooled QC volumes; features were retained for further analysis if they exhibited a significant positive correlation (p ≤ 0.05), i.e., a linear signal response to increased amount injected. Dispersion ratio (a comparison between signal variance in pooled QC replicate injections versus samples) was used to assess variance; only features with a value below 50 were retained for further analysis (Broadhurst et al., 2018).

Feature abundances were log₂-transformed to stabilize variance and then median-normalized across samples. Median normalization applies a linear shift to each sample, aligning the median log fold change (logFC) to zero without altering the relative distribution of signal within samples. This method assumes that the majority of features remain unchanged across experimental conditions. Detection criteria required a feature to be present in at least two-thirds of samples within at least one experimental group (JDM/DM, HC, or US). Missing values were excluded. Data visualization was used to assess sample quality, variance, and clustering. Heatmaps were generated using the ComplexHeatmap package (version 2.15.4) for manual inspection of clustering patterns and identification of potential outliers. Principal component analysis (PCA) was performed to evaluate sample distribution and detect outliers. MA plots were used to verify that the median normalization procedure aligned the bulk of data to y = 0 and that feature abundance was normalized across the full signal range. Within-sample variance was assessed using the median absolute deviation (MAD). Metabolite class annotations were assigned using the classyfireR package.

Statistical analysis was conducted using a custom R workflow (R version 4.3.1) built around the limma package (version 3.56.2). To account for the matched study design, trio number—representing one affected subject (JDM or DM), one US, and one sex- and age-matched HC—was included as a blocking factor. P-values were adjusted for multiple testing using the Benjamini–Hochberg method. Welch’s two-sided t-tests were performed on the numerical sample metadata to identify significant differences across groups. HCs were enrolled in the study after JDM and US enrollment, therefore the sample collection year was a known significant difference between JDM/ US and HC. The samples did not appreciably resolve by collection year based on visual inspection of PCA, MA-plots, and heatmaps after normalization. Including sample collection year as a covariate in the statistical model did not appreciably alter the results and was therefore excluded from the final model. This decision was further supported by the paired study design, in which within-pair dependencies were accounted for using the trio identifier as a blocking factor. To identify associations between metabolite abundances and sample metadata, correlation analyses were performed. Normality was assessed using the Shapiro–Wilk test to determine the appropriate correlation method (Spearman or Pearson). For the reported analyses, Spearman correlation was used to evaluate associations between feature abundances and sample metadata. For both differential and correlation analysis, metabolites with adjusted p-values < 0.1 were considered for interpretation though adjusted p-values < 0.05 should be considered higher confidence. This less stringent threshold was used to prioritize identification of potentially meaningful patterns rather than strict control of false positives.

## Results

### Multivariate analysis

**Fig. 1:**
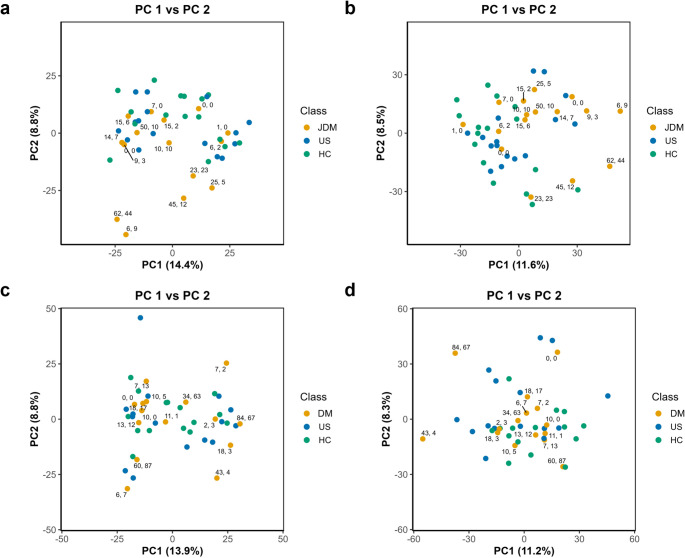
Multivariate analysis via PCA of JDM or DM (yellow) from US (blue) and HC (green). PCA score plots for JDM cases and controls in **a** positive and **b** negative ionization modes. PCA score plots for DM cases and controls in **c** positive and **d** negative ionization modes. JDM/DM points annotated with Physician Global Activity, Physician Global Damage scores

To investigate metabolism changes in the serum of JDM and DM patients, we analyzed serum samples from 16 JDM patients and 15 DM patients, each matched with an unaffected same-sex, closest-in-age US sample and a same-sex, age-matched HC sample. Detailed information on study subjects is displayed in Table 1. Aside from the difference in sample collection year addressed in the Methods section, no notable differences in sample metadata were observed among the JDM contrasts. Two potentially meaningful differences were identified within the adult DM group: alanine transaminase (ALT) levels were significantly higher in DM subjects compared to HC and alkaline phosphatase (ALP) levels were significantly lower in DM subjects compared to US. Note that there was not full coverage of measurements across all samples. Following data quality filtering, 3660 metabolite features in positive ionization mode and 5467 features in negative ionization mode remained for subsequent statistical analyses. The overall serum metabolomic profile, evaluated via principal component analysis (PCA), did not significantly differentiate JDM, US and HC samples (Fig. 1a, b) or DM, US and HC samples (Fig. 1c, d). Notably, JDM patients with the highest Physician Global Activity (PGA) and Physician Global Damage (PGD) scores separated from control groups and most lower disease activity and damage JDM patients (Fig. 1a, b).

### Significant differences in serum metabolites between JDM, unaffected siblings, and unrelated healthy controls

Differential analysis was performed using limma to compare combinations of patient, US, and HC classes within the JDM and DM groups, with trio identifier included as a blocking factor. The blocking factor captures the paired design and helps adjust for baseline characteristics by design, particularly through the US sibling comparison, which accounts for shared familial and environmental factors that are often difficult to capture with other measures. Comparison of JDM patient serum to US and HC resulted in 247 and 656 differential features, respectively. Comparison of DM patient serum to US and HC serum resulted in 52 and 169 differential features, respectively. The statistical output for all features and comparisons is available in Supplementary Table S2. Given the untargeted metabolomics approach utilized here, a small portion (< 20%) of the significant features were annotated with putative chemical names. In order to focus on biochemical insights, only metabolites with putative or confirmed identifications are discussed herein. All metabolites differentiating DM and US serum were putatively annotated as steroids or steroid-like metabolites (Supplementary Fig. S1), consistent with corticosteroid treatment (Supplementary Table S1) (Bohan & Peter, 1975b). This was not the case for differential metabolites arising from the JDM versus US comparison, which were primarily non-steroidal. Therefore, JDM is the focus of this work, with particular interest in metabolites differentiating JDM and US groups. Further details on the statistical analysis and annotation of the metabolites of interest are provided in Supplementary Table S3 and Supplementary Fig. S2–S17.

**Fig. 2:**
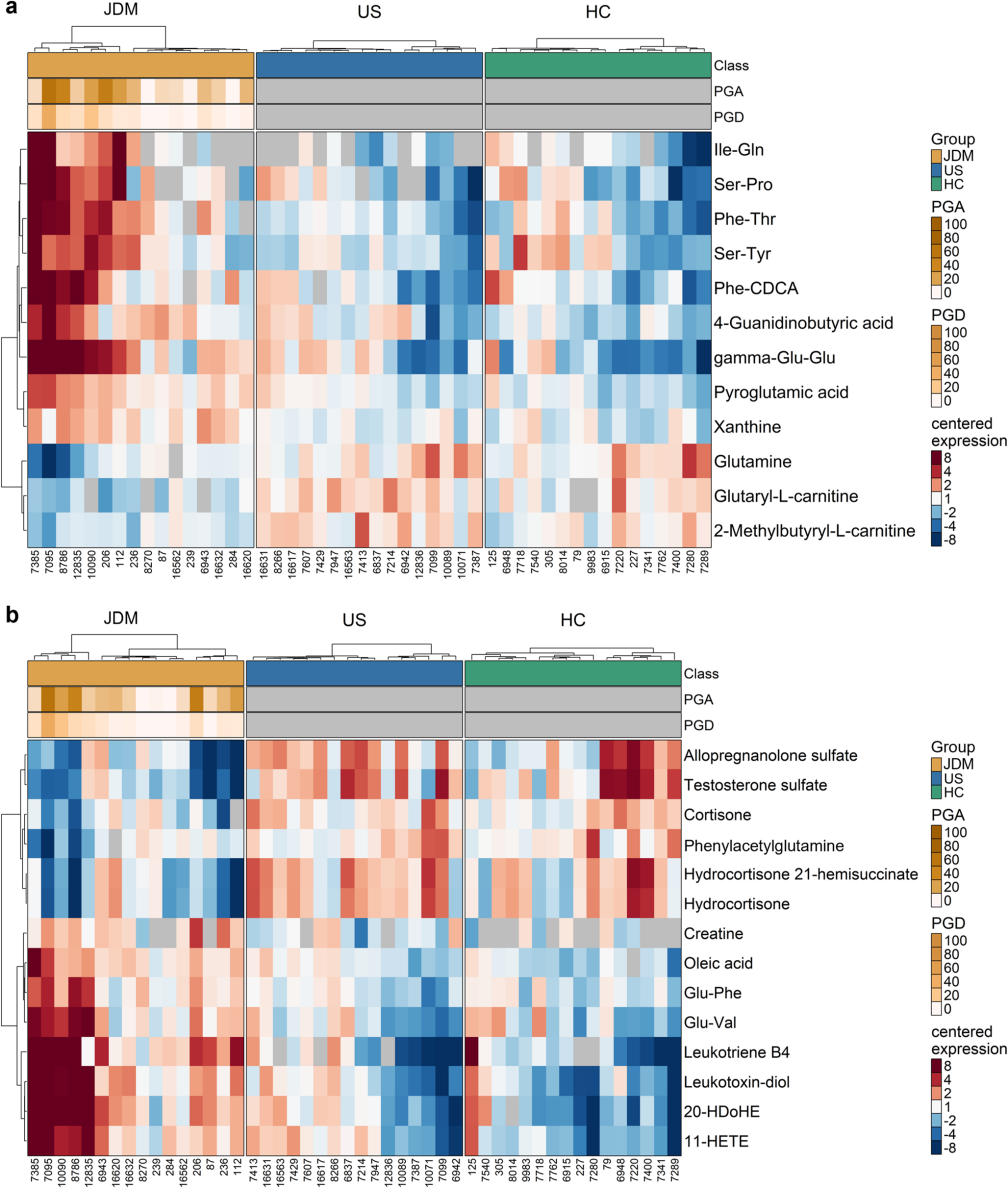
Differential serum metabolite profiles of JDM, US and HC groups. Heatmaps of annotated differential serum metabolites between JDM and US groups in **a** positive and **b** negative ionization modes

Heatmaps of all annotated significant metabolites demonstrate their differential abundances in JDM, US and HC groups (Fig. 2). JDM patients with lower PGA and PGD scores had metabolite abundances more similar to US and HC serum than JDM patients with greater disease activity and damage.

**Fig. 3:**
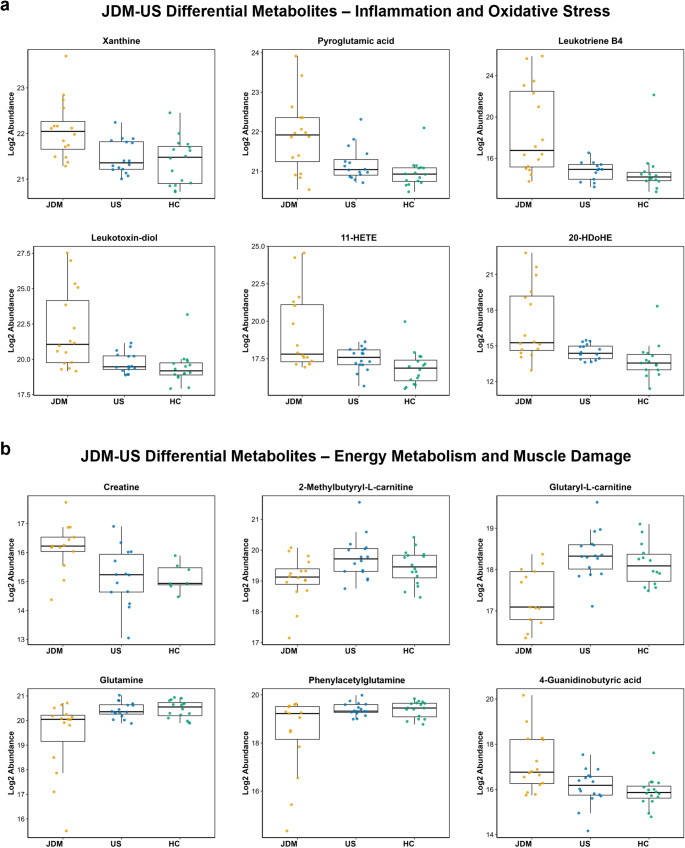
Differential serum metabolites of interest in JDM compared to US and HC groups. Box plots of differential metabolites in JDM vs. US and HC. Metabolites grouped by relation to **a** inflammation and oxidative stress or **b** energy metabolism and muscle damage

Many significant differences between JDM and US serum were metabolites related to two major categories, inflammation and oxidative stress (Fig. 3a) or energy metabolism and muscle damage (Fig. 3b). The levels of the purine xanthine and the amino acid derivative pyroglutamic acid were significantly increased in JDM patients compared to both US (FC = 1.5, adj. p-value = 0.04) and HC (FC = 1.6, adj. p-value = 0.007) groups. Several bioactive lipid features, including putative eicosanoids and pro-resolving lipid mediators, were also increased in the JDM patient serum compared to US and HC. These features were putatively annotated as leukotriene B4 (5,12-diHETE), leukotoxin-diol (9,10-diHOME), 11-HETE, and 20-HDoHE at MSI levels 3 or 4, representing their lipid mediator subclasses rather than definitive metabolite identities. For example, the MS/MS-based annotation of 11-HETE should be interpreted as a constituent metabolite/s from the HETE/EET bioactive lipid class based on the shared *m/z,* shared product ions (e.g. *m/z* 167), and lack of retention time information.

The levels of the energy metabolism and muscle-related metabolite creatine were increased in JDM patients compared to US and HC; however, this difference was only statistically significant in the JDM versus US comparison (FC = 2, adj. p-value = 0.09). Two acylcarnitines, 2-methylbutyryl-L-carnitine and glutaryl-L-carnitine, as well as the amino acids glutamine and phenylacetylglutamine were significantly decreased in JDM patients compared to US and HC groups. Levels of the alkaloid 4-guanidinobutyric acid (GBA) were increased in JDM patients compared to both control groups. Fold changes and adjusted p-values for all metabolites of interest are provided in Supplementary Table S3.

### Correlation analysis reveals metabolite trends related to JDM physician global damage scores

**Fig. 4:**
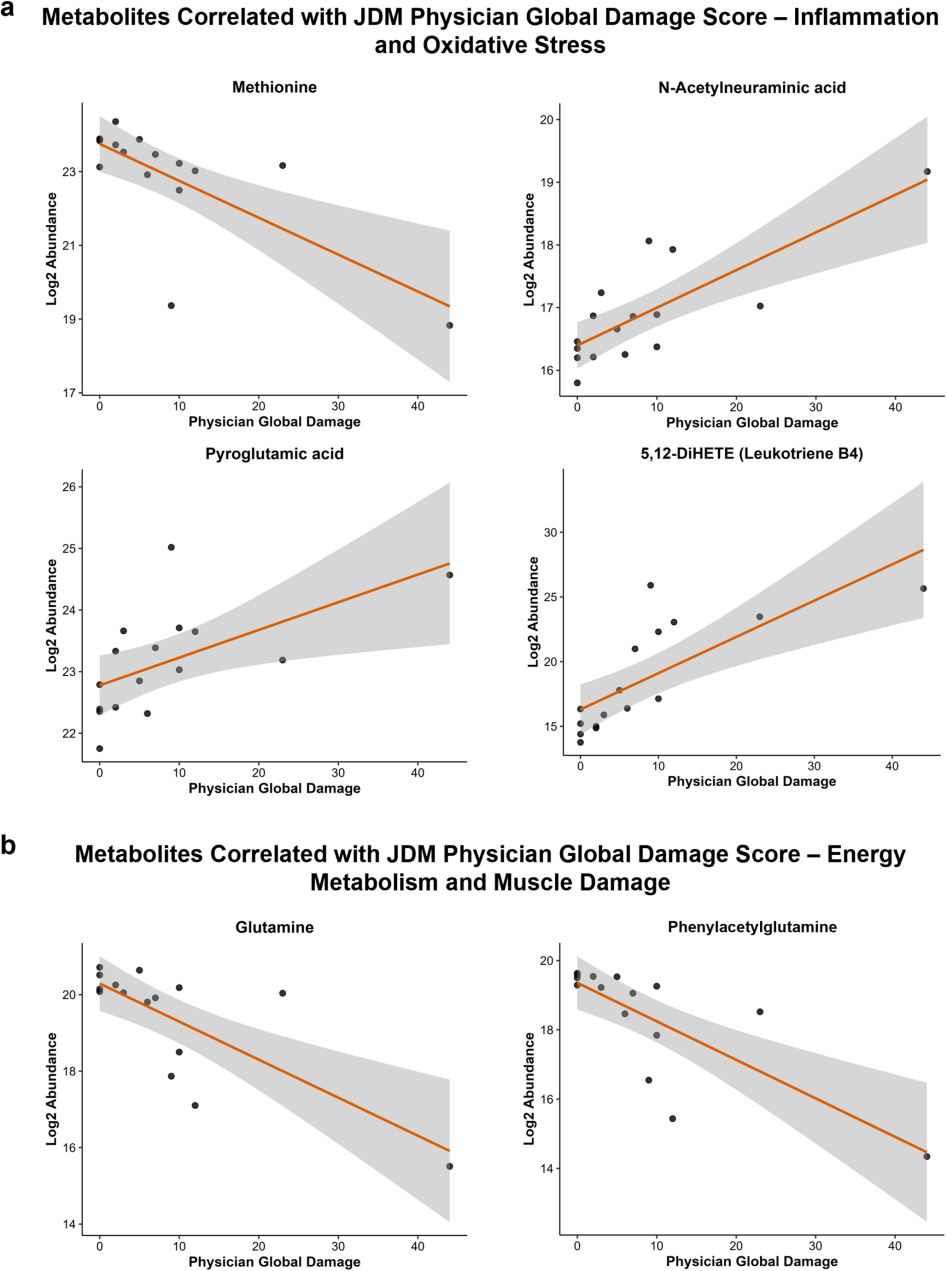
Correlations between Physician Global Damage scores and serum metabolite levels in JDM. Correlation plots displaying the relationship between Physician Global Damage and metabolite levels in JDM patients. Metabolites related to **a** inflammation and oxidative stress or **b** energy metabolism and muscle damage are displayed

Given the observation of more pronounced differential metabolite abundances in JDM patients with higher PGA and PGD scores (Fig. 2), we analyzed the correlation between feature abundance and disease activity/damage in all JDM patients. We considered correlations with an adjusted p-value below 0.10 for interpretation, while correlations with an adjusted p-value below 0.05 should be regarded as higher confidence. The full correlation output can be found in Supplementary Table S4. While both PGA and PGD scores were evaluated in addition to other numeric clinical variables, none of the named metabolites correlated significantly with PGA scores. The trends observed between metabolite abundance and PGD score were shared with PGA since the relative scores were similar for each patient (Fig. 2), but PGD is the only clinical measure of disease status discussed hereafter. Several metabolites correlated with PGD scores were also related to either inflammation and oxidative stress (Fig. 4a) or energy metabolism and muscle damage (Fig. 4b). The amino acid methionine was negatively correlated with disease damage, whereas the abundance of a tentatively identified sialic acid (N-acetylneuraminic acid) was positively correlated with disease damage. Many metabolites were both differential between JDM patients and control groups as well as correlated with PGD scores, including pyroglutamic acid and several bioactive lipids (Fig. 4a, Supplementary Fig. S18), which were all increased in JDM patients versus controls and positively correlated with disease damage scores. Conversely, glutamine and phenylacetylglutamine were decreased in JDM patients compared to controls and negatively correlated with PGD scores (Fig. 4b). No identified metabolites were significantly correlated with DM patient PGA or PGD scores.

## Discussion

Here, we performed untargeted metabolomics analysis to identify metabolite changes in juvenile and adult dermatomyositis patient serum compared to age- and sex-matched unaffected siblings and healthy controls. Multivariate, univariate, and correlation analyses revealed that some metabolome changes in JDM serum were associated with clinical measures of disease status, primarily measured by Physician Global Damage (PGD) scores as well as Physician Global Activity (PGA) scores. While PGA scores reflect the patient inflammatory disease activity at the time of sample collection, PGD scores are a measure of the cumulative consequences of the disease and treatment. JDM patients with the highest PGD and PGA scores were distinguished from controls by PCA, however no significant differentiation of sample groups was observed (Fig. 1). A gradient response based on condition, rather than major metabolomic profile differences between groups, is unsurprising for serum samples from patients undergoing treatment. In absence of strong global separation in PCA, individual differential metabolites and those correlated with PGD scores are the focus of this discussion. Although correlation patterns with PGA and PGD scores were consistent (Fig. 2), statistically significant (adj. p-value < 0.1) associations were only observed with PGD. This is likely due to the cross-sectional study design, treatment suppression of active inflammation, and limited, positively skewed distribution of PGA scores (median = 11, mean = 20, max = 84). Thus, the correlation analysis results are more reflective of metabolites associated with accumulated disease consequences, physical deconditioning, and treatment exposure rather than transient inflammatory activity.

The major focus of this work is on comparing JDM and US groups to highlight changes unrelated to genetic and environmental differences. Notably, most identified differential metabolites when comparing JDM to US were also significant when comparing JDM to HC. The reverse was not observed, as many metabolites were significant when comparing JDM to HC but not to US (Table S2). This is reflected in the relative abundance levels of metabolites in each sample group, where the abundance in US serum is often intermediate between that in HC and JDM (Figs. 2, 3). Despite the similarities in JDM and DM, our study did not detect significant serum metabolite changes in DM patients other than those directly linked to corticosteroid treatment. Treatment effects, greater disease heterogeneity, and uncontrolled environmental and lifestyle factors unique to adults may contribute to the lack of significant differences observed in DM. A previous longitudinal JDM study reported declining metabolic differences with treatment (Dvergsten et al., 2022).

Differential analysis was based on relative feature abundances rather than absolute metabolite quantities. False discovery adjustment was performed using the Benjamini–Hochberg method, and metabolites with an adjusted p-value below 0.10 were prioritized for interpretation as hypothesis-generating signals in this exploratory untargeted dataset. A targeted assay would be better suited to confirm and better resolve specific differences, since untargeted metabolomics prioritizes breadth of coverage and can have lower sensitivity and higher feature-level variability for individual metabolites. Findings with p-values below 0.05 should be regarded with higher confidence as they have a lower risk of false positives, though there were few notable metabolites at this threshold (Supplementary Table S2). Several quality control steps (detailed in the Methods section) were taken to minimize technical variability such that relative comparisons of feature abundances could be attributed to biological differences. However, future targeted quantitative validation of the differential metabolites is required to evaluate absolute concentration changes and confirm metabolite identities.

Other major limitations of the present study include the small patient cohort (n = 16 for JDM and n = 15 for DM) leading to minimal statistical power when thousands of features are evaluated, and limited metabolite annotations, a common bottleneck in untargeted metabolomic studies (Gertsman & Barshop, 2018). The paired design is a key strength of this study and helps mitigate major baseline and shared familial or environmental confounding. However, inference is driven by within-trio contrasts and extensive covariate adjustment is not well supported given the small number of trios. Sample size and sparse autoantibody positive coverage (e.g., anti-MDA5, anti-TIF1γ, anti-NXP2), compounded by the paired design, constrained phenotype stratified comparisons; these analyses would be appropriate to pursue in a larger cohort, but are a limitation of the present study. Accordingly, findings should be interpreted as reflecting SRD status in the context of real-world clinical management, and residual confounding, particularly related to treatment exposure and timing, cannot be fully excluded.

Many metabolomic changes identified in JDM patient serum were indicative of inflammation and oxidative stress (Fig. 3a**, **Fig. 4a). Xanthine and pyroglutamic acid were both increased in JDM, with pyroglutamic acid also positively correlated with PGD scores. Elevated xanthine levels can increase oxidative stress, as xanthine metabolism produces reactive oxygen species (ROS) and the pro-inflammatory end product uric acid. Uric acid itself, measured both as a clinical variable in some samples (Table 1) and as a serum metabolite by LC–MS, was not found to be significantly different between any sample groups or correlated with disease damage. Previous studies have reported differential xanthine levels in PM patient plasma and increased abundance of its precursor hypoxanthine in DM patient plasma (Zhao et al., 2023). Increased serum pyroglutamic acid may be indicative of disruptions in the γ-glutamyl cycle and subsequent depletion of the antioxidant glutathione. No reports of this finding in DM patients were identified. However, patients with systemic lupus erythematosus (SLE) were shown to have both increased pyroglutamic acid and decreased glutathione in their serum (Wu et al., 2012; Zhang et al., 2021). These findings suggest shared pathways of oxidative stress between SARDs. Interestingly, pyroglutamic acid was reportedly downregulated in calcified human skeletal muscle cells. These cells exhibited elevated mitochondrial oxidative stress and compromised oxidative capacity (Duvvuri et al., 2023). Methionine also contributes to antioxidant defense as a precursor to cysteine and, therefore, glutathione. Methionine was proposed as one of several metabolite biomarkers of DM by Zhang et al. as it was decreased in DM patients compared to controls (Zhang et al., 2019). Methionine was negatively correlated with PGD scores although it was not significantly differential between JDM and controls in this study. Another signature of inflammation is sialic acid-binding immunoglobulin-like lectin 1 (Siglec-1), a strong interferon-inducible marker that mediates macrophage-pathogen adhesion. Siglec-1 is a promising biomarker for chronic immune-mediated inflammatory diseases and has been shown to correlate with disease activity in JDM (Lerkvaleekul et al., 2022). Siglec-1 binds sialic acids, and the putative sialic acid feature N-acetylneuraminic acid was negatively correlated with disease damage scores, possibly indicating a diminished availability of these ligands.

Bioactive lipids play a crucial role in immune regulation and inflammation by modulating the activity of immune cells and production of inflammatory cytokines. Therefore, prolonged overexpression of bioactive lipids is a hallmark of chronic inflammation such as muscle inflammation in DM. We observed several putative bioactive lipids with increased abundance in JDM compared to controls that were also positively correlated with JDM disease damage. Leukotriene B4 is an eicosanoid derived from arachidonic acid through the lipoxygenase pathway. As a potent pro-inflammatory mediator and well-studied bioactive lipid, the leukotriene pathway has been investigated for its involvement in DM and PM. These studies report upregulation of the leukotriene pathway in muscle tissue from patients with DM and PM and suggest that leukotriene B4 has roles in sustaining inflammation and muscle weakness (Loell et al., 2010, 2013). It is important to note that lipid (e.g., steroid and lipid mediator) annotations are more difficult to validate with the untargeted metabolomics method used for this study. While the identifications are putative with lower confidence scores (MSI level 3/4), the chemical classification of these lipid features is of high confidence. Future targeted studies evaluating specific bioactive lipids are warranted, especially leukotriene B4 given its relevance to DM.

Several metabolites related to energy metabolism and muscle damage were also impacted by JDM diagnosis and disease damage (Fig. 3b**, **Fig. 4b). Creatine levels were increased in JDM serum compared to controls indicating increased energy demand and muscle tissue damage. The conversion of creatine to phosphocreatine, catalyzed by creatine kinase (CK), is the primary energy reservoir for muscles. Serum CK levels are typically elevated in DM and used as a marker of DM treatment efficacy (Iorizzo & Jorizzo, 2008). Chung et al. found an increased creatine to creatinine ratio (Cr:Cn) in juvenile IIM urine relative to controls, and that Cr:Cn correlated positively with Physician Global Disease Damage scores (Chung et al., 2005). Increased plasma creatine was observed previously in patients with various IIMs, specifically those within anti-Mi2 + and anti-SRP + autoantibody subtypes (Liu et al., 2022). Another guanidino compound, 4-guanidinobutyric acid (GBA), had lower abundance in JDM serum compared to controls. GBA is a byproduct of creatine synthesis from arginine. Further studies to determine its specific role in energy metabolism and potential as a muscle damage marker are needed (Sinn et al., 2022; Tachikawa & Hosoya, 2011). Glutamine can be synthesized and stored in muscle tissue and plays an important role in muscle homeostasis and inflammatory response to muscle damage (Rennie et al., 1989; Shang et al., 2020). In JDM patients, glutamine levels were lower than controls and correlated negatively with PGD scores, indicating increased metabolic demands of chronic inflammation and energy production needed for muscle repair. Previous studies also reported a significant decrease in glutamine in DM patient serum (Zhang et al., 2019). The same trend was observed for the gut microbiota-related glutamine derivative phenylacetylglutamine. This may be indicative of general microbiome alterations in JDM patients, as decreased microbial diversity and changes in taxonomic composition have been demonstrated in DM patients (Bae et al., 2022). Once formed, the highly nitrogenous metabolite phenylacetylglutamine plays a role in nitrogen excretion as an alternative to the urea cycle (Brusilow, 1991). As urea cycle activity is increased with muscle breakdown, the need for alternative nitrogen disposal routes decreases. Phenylacetylglutamine in blood was found to be a protective metabolite in the development of the SARD rheumatoid arthritis (Yu et al., 2022). Two acylcarnitines (ACs) were decreased in JDM serum compared to controls. ACs are involved in fatty acid β-oxidation and energy production. Changes in ACs can indicate dysfunction of mitochondrial fatty acid β-oxidation. This result is consistent with findings of major AC dysregulation in JDM serum; however, the AC chain length and trends differed from the current study (Dvergsten et al., 2022).

## Conclusions

Our untargeted metabolomics analysis highlights metabolic disruptions underlying JDM, particularly related to inflammatory and energy processes. Metabolites that were differential in JDM serum compared to US and correlated with physician global damage (PGD) scores, including pyroglutamic acid, bioactive lipids, glutamine, and phenylacetylglutamine, represent promising metabolic signatures associated with disease burden while accounting for genetic backgrounds. There were no differential metabolites of interest when comparing DM to US serum, as the only named significant features were attributed to corticosteroid treatment. This study was limited by small sample size and by the limited ability to annotate metabolite features, a common challenge for discovery-based untargeted metabolomics. Additionally, several myositis autoantibody subgroups were included within the JDM and DM patient groups, as there were not enough patients in any one autoantibody subgroup to investigate them separately. Future targeted, quantitative studies from a larger study population would be useful to validate the observed metabolite changes. Despite these limitations, the results reported here highlight serum metabolite changes in JDM which should be explored in further studies.

## Supplementary Information

Below is the link to the electronic supplementary material.

Supplementary file1 (XLSX 6093 KB)

Supplementary file2 (DOCX 5847 KB)

## Data Availability

De-identified raw untargeted metabolomics data generated and analyzed during the current study is available in the MASSIVE repository (MSV000098749) with restricted access due to patient confidentiality. Access available upon request.
