# Supplementary material for: Untargeted metabolomics reveals serum metabolites related to energy metabolism and inflammation associated with juvenile dermatomyositis
Source: Metabolomics. 2026 Apr 29;22(3):60. doi: 10.1007/s11306-026-02425-5 (PMC13124813; doi:10.1007/s11306-026-02425-5)
Supplement: Supplementary file 2 — Supplementary file2 (DOCX 5847 KB) [file 11306_2026_2425_MOESM2_ESM.docx]

Supplementary Materials for

**Untargeted Metabolomics Reveals Serum Metabolites Related to Energy Metabolism and Inflammation Associated with Juvenile Dermatomyositis**

Kaylie I. Kirkwood-Donelson^1§^, Dylan J. Johnson^2§^, Payam Noroozi Farhadi^3^, Kakali Sarkar^3^, Adam I. Schiffenbauer^3^, Frederick W. Miller^3^, Jian-Liang Li^2^, Lisa G. Rider^3^, Alan K. Jarmusch^1^

^1^Metabolomics Core Facility, Immunity, Inflammation and Disease Laboratory,

^2^Integrative Bioinformatics Support Group, Biostatistics and Computational Biology Branch,

^3^Environmental Autoimmunity Group, Clinical Research Branch,

National Institute of Environmental Health Sciences, National Institutes of Health, Durham, NC, USA

**Table of Contents**

**Table S1** (excel tab Table.S1): Univariate statistical output for all features

**Table S2** (excel tab Table.S2): Statistics and correlation values for metabolites of interest

**Table S3** (excel tab Table.S3): Correlation output for all features

**Fig. S1** (pg 2): Heatmap of annotated differential serum metabolites

**Fig. S2 – 17** (pg 3-11): MS and MS/MS spectra for metabolites of interest

**Fig. S18** (pg 12): Correlation of additional bioactive lipids with PGD


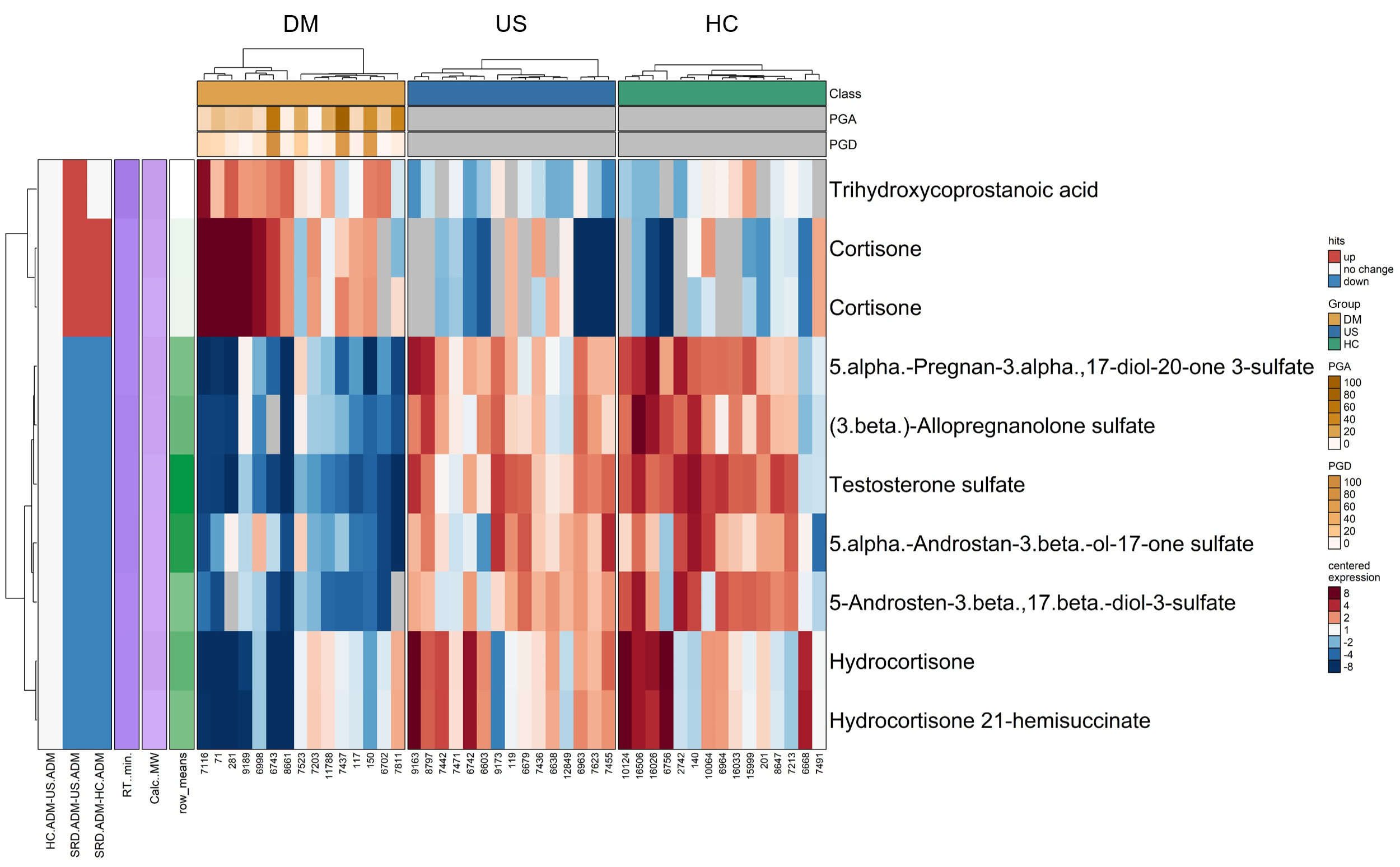


**Fig. S1.** Heatmaps of annotated differential serum metabolites between dermatomyositis, unaffected sibling, and healthy control groups.


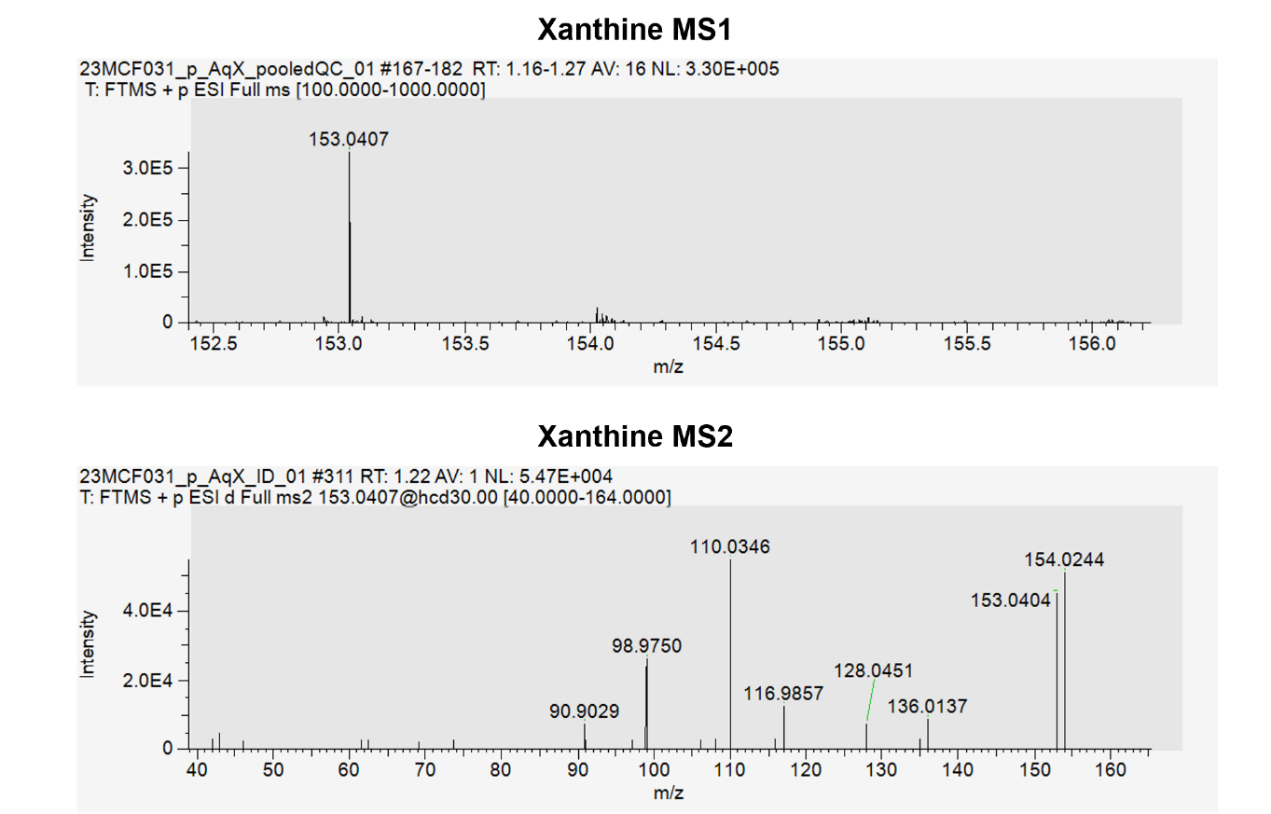


**Fig. S2.** Representative spectra for feature annotated as xanthine (level 2) in positive mode.


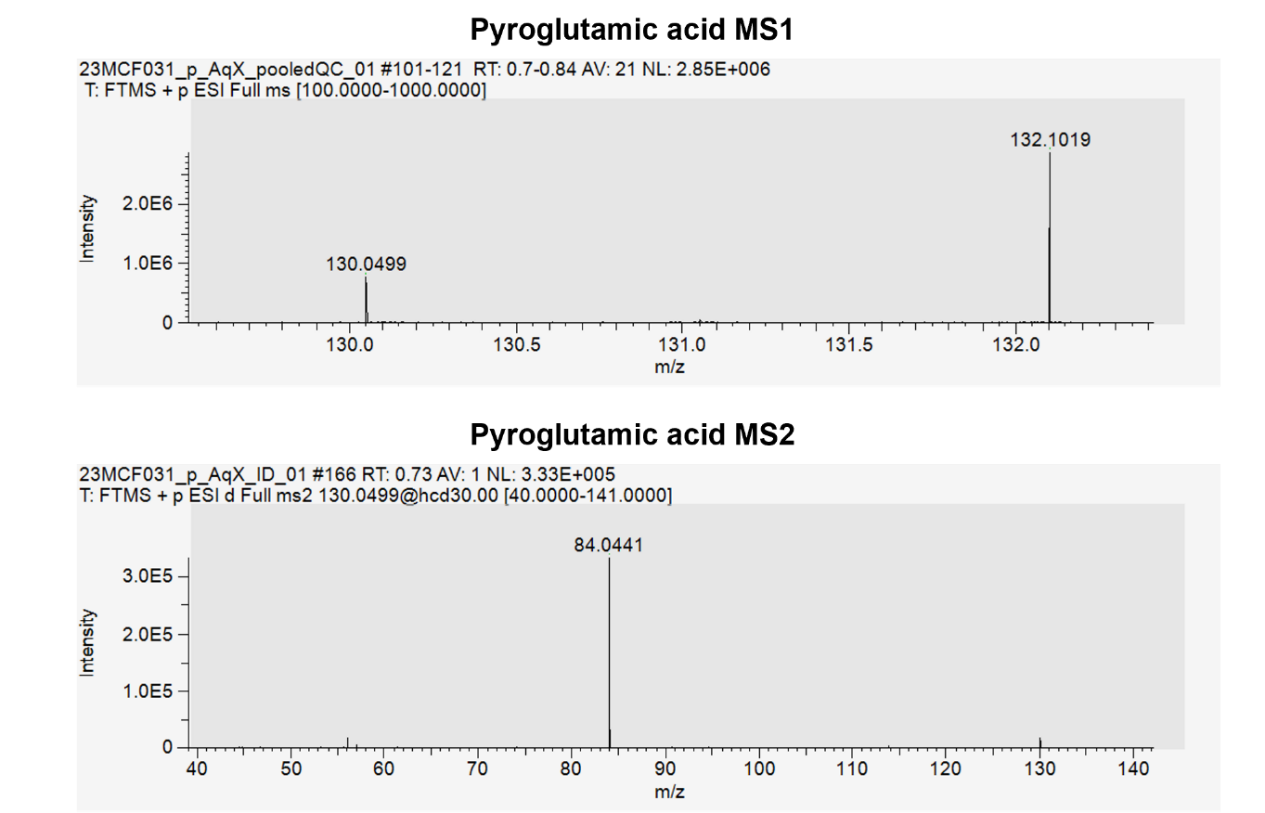


**Fig. S3.** Representative spectra for feature annotated as pyroglutamic acid (level 1) in positive mode


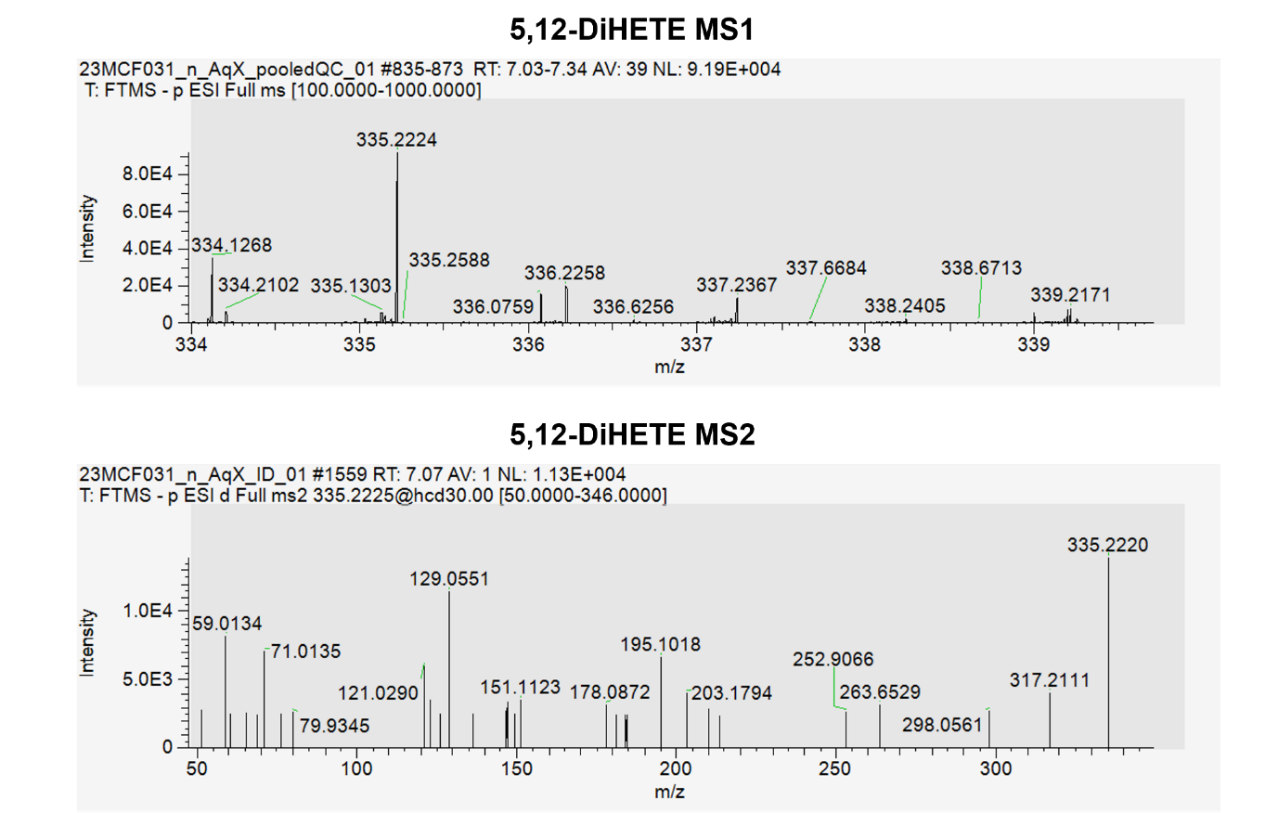


**Fig. S4.** Representative spectra for feature annotated as 5,12-DiHETE or leukotriene B4 (level 3) in negative mode.


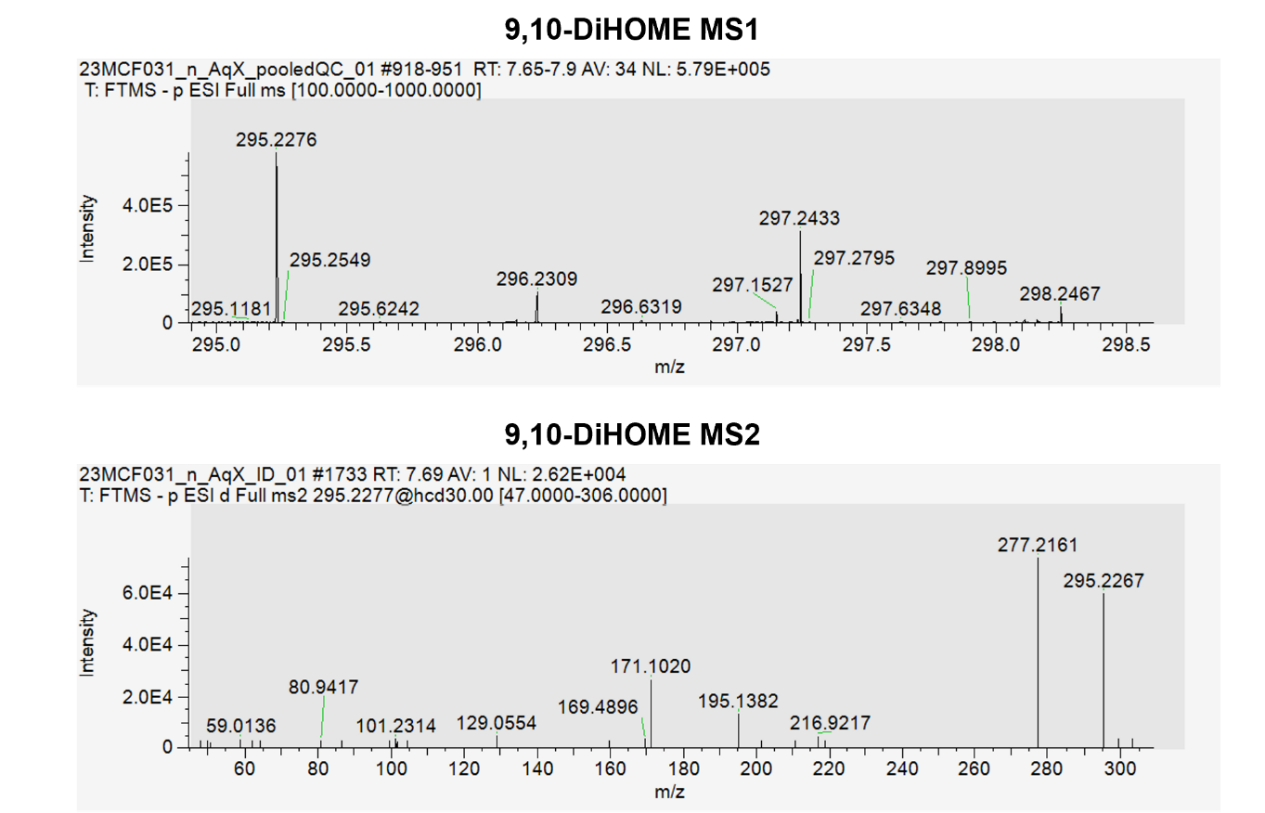


**Fig. S5.** Representative spectra for feature annotated as 9,10-DiHOME or leukotoxin diol (level 4) in negative mode.


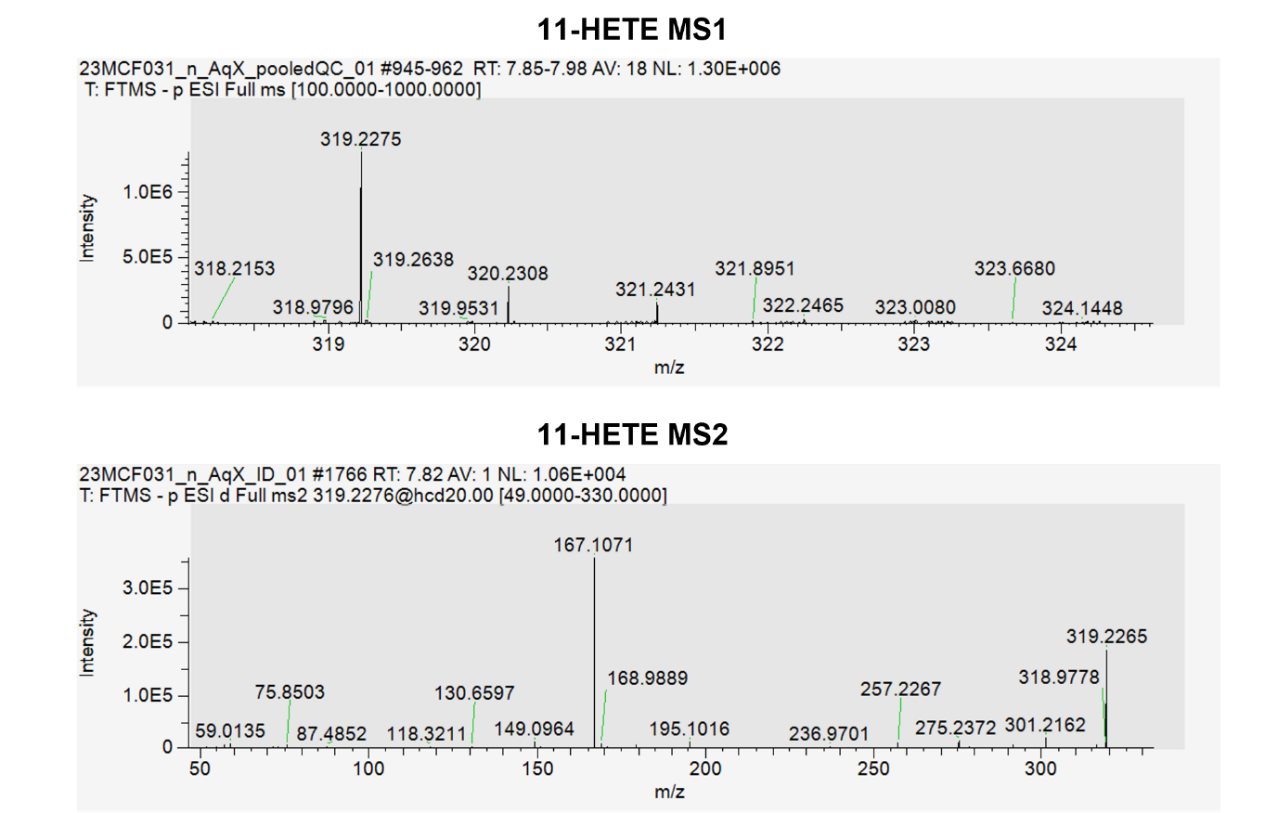


**Fig. S6.** Representative spectra for feature annotated as 11-HETE (level 3) in negative mode.


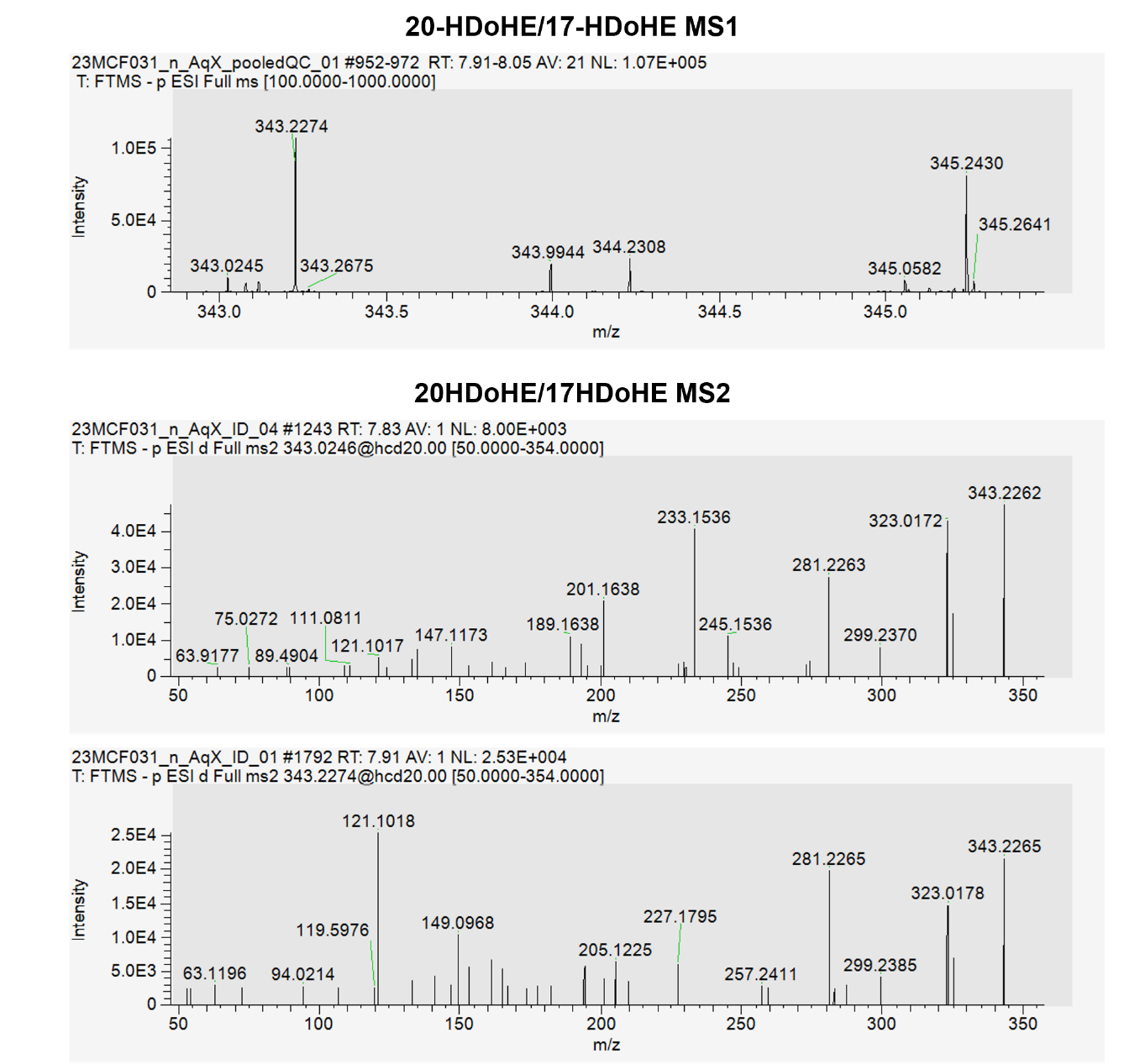


**Fig. S7.** Representative spectra for feature annotated as the isomers 20-HDoHE and 17-HDoHE (level 3) in negative mode.


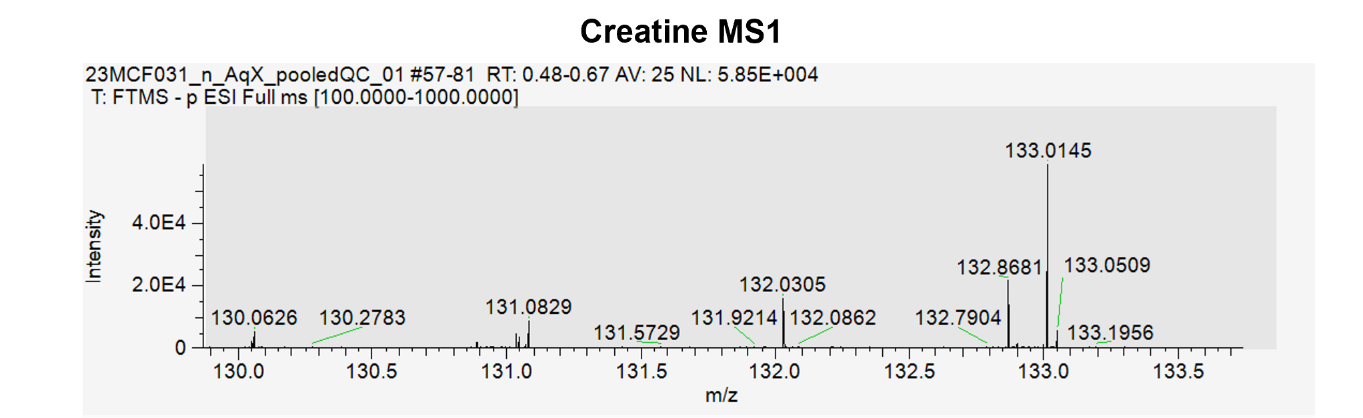


**Fig. S8.** Representative spectra for feature annotated as creatine (level 3) in negative mode. This feature was not selected for MS/MS.


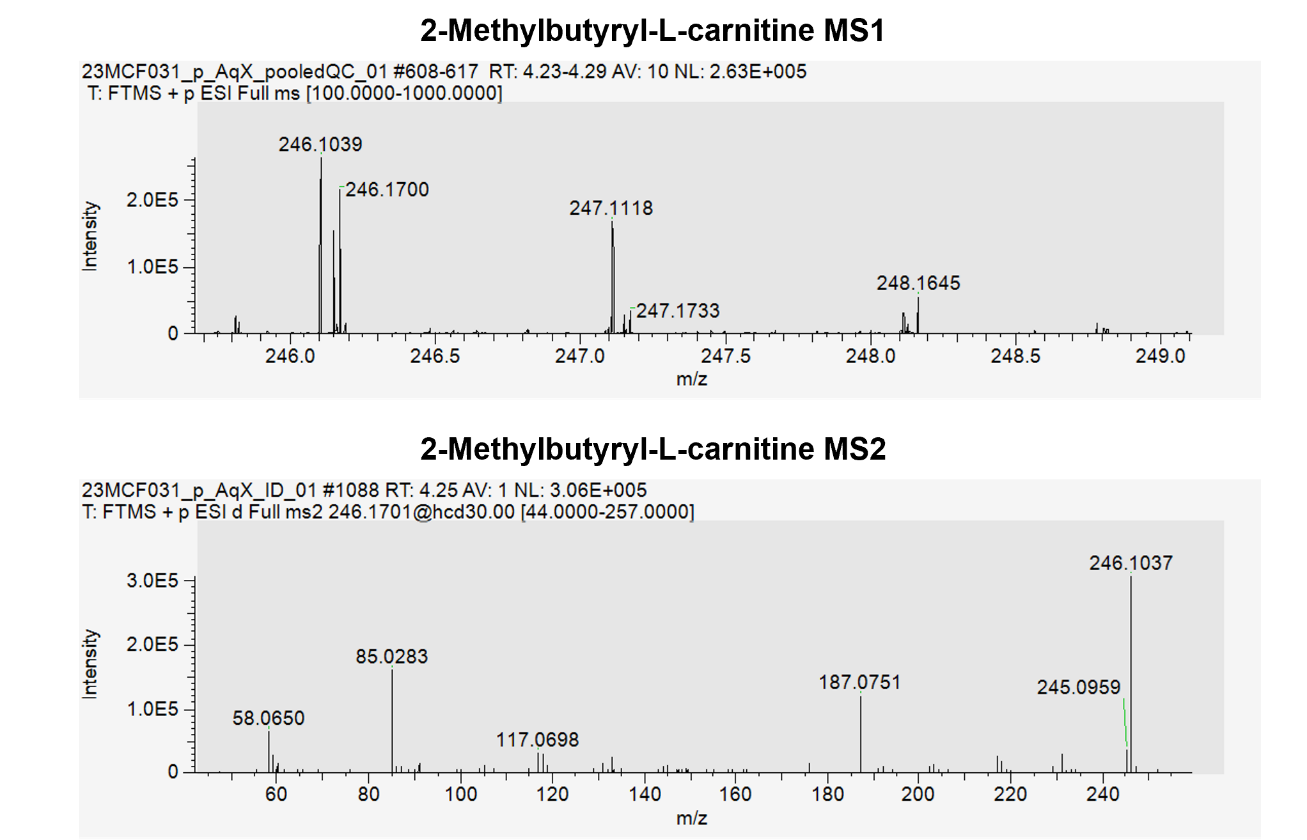


**Fig. S9.** Representative spectra for feature annotated as 2-methylbutyryl-L-carnitine (level 2) in positive mode.


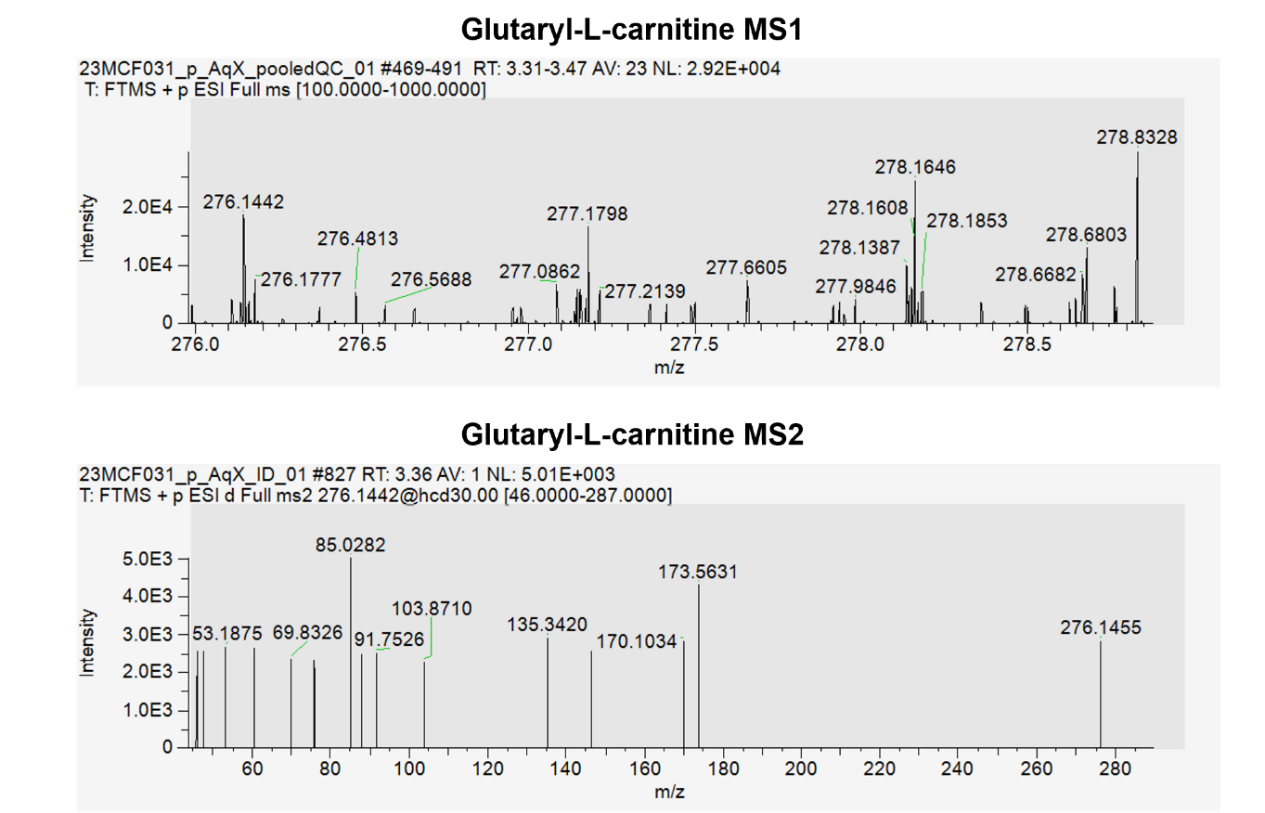


**Fig. S10.** Representative spectra for feature annotated as glutaryl-L-carnitine (level 1) in positive mode.


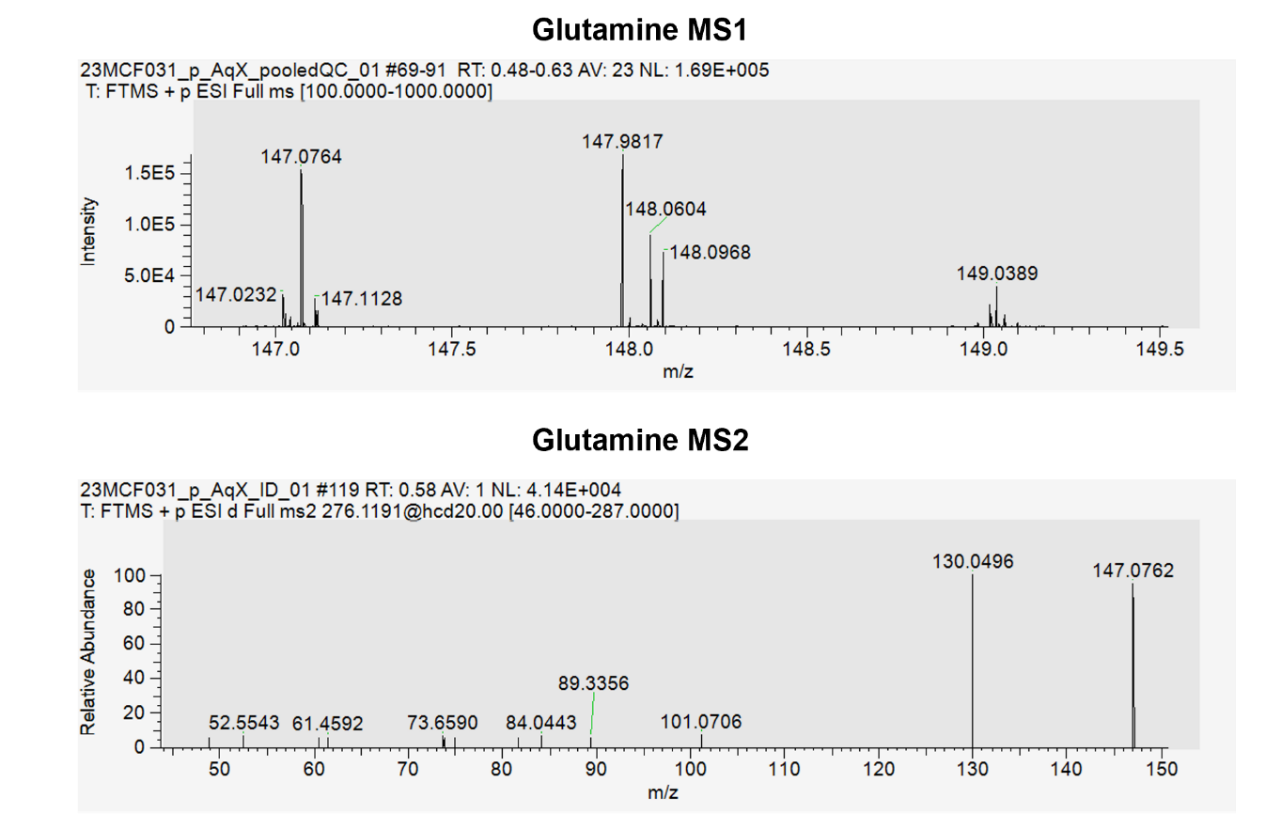


**Fig. S11.** Representative spectra for feature annotated as glutamine (level 1) in positive mode.


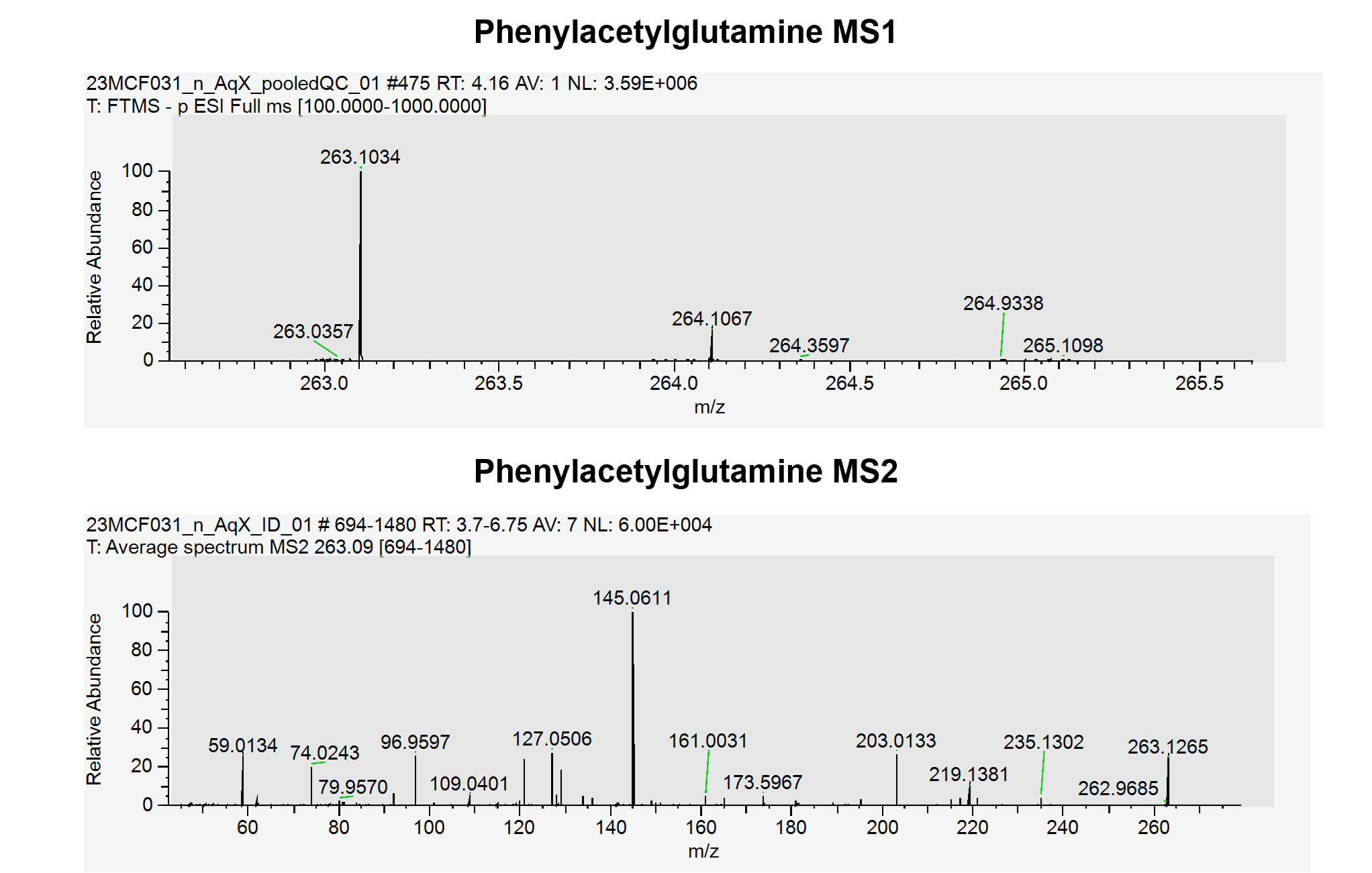


**Fig. S12.** Representative spectra for feature annotated as phenylacetylglutamine (level 2) in negative mode.


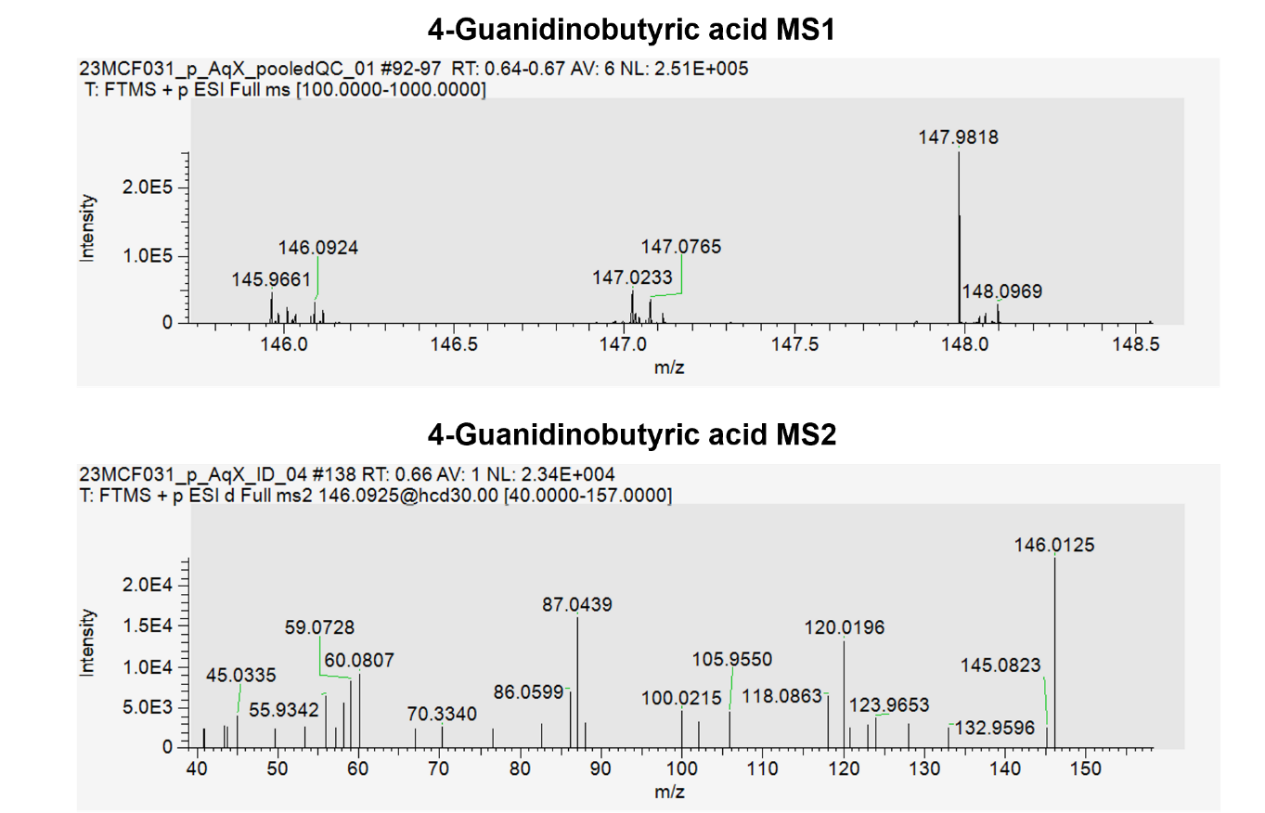


**Fig. S13.** Representative spectra for feature annotated as 4-guanidinobutyric acid (level 1) in positive mode.


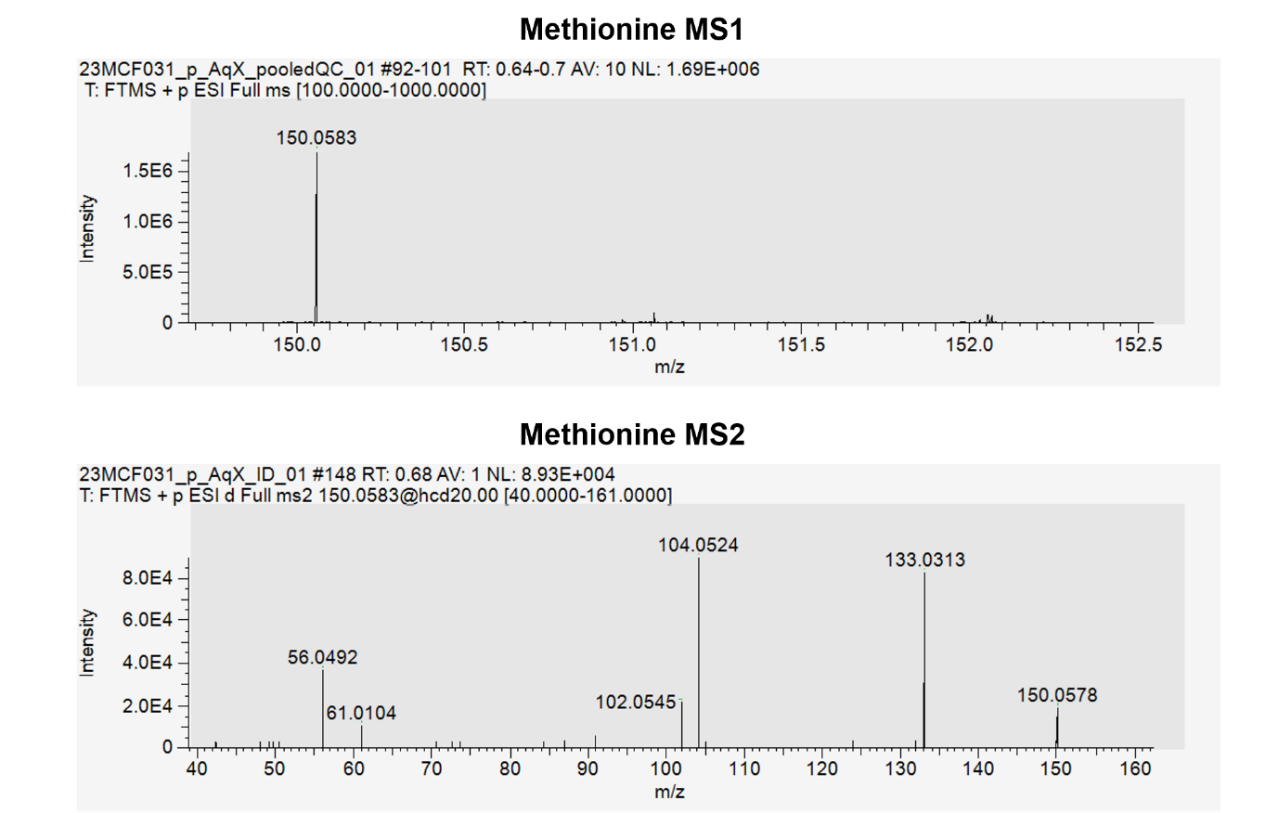


**Fig. S14.** Representative spectra for feature annotated as methionine (level 1) in positive mode.


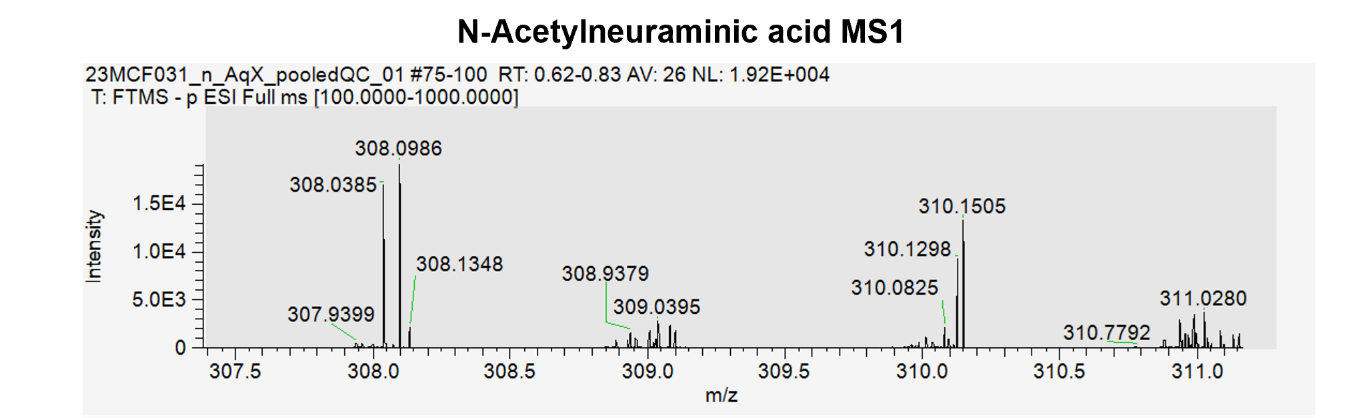


**Fig. S15.** Representative spectra for feature annotated as N-acetylneuraminic acid (level 3) in negative mode. This feature was not selected for MS/MS.


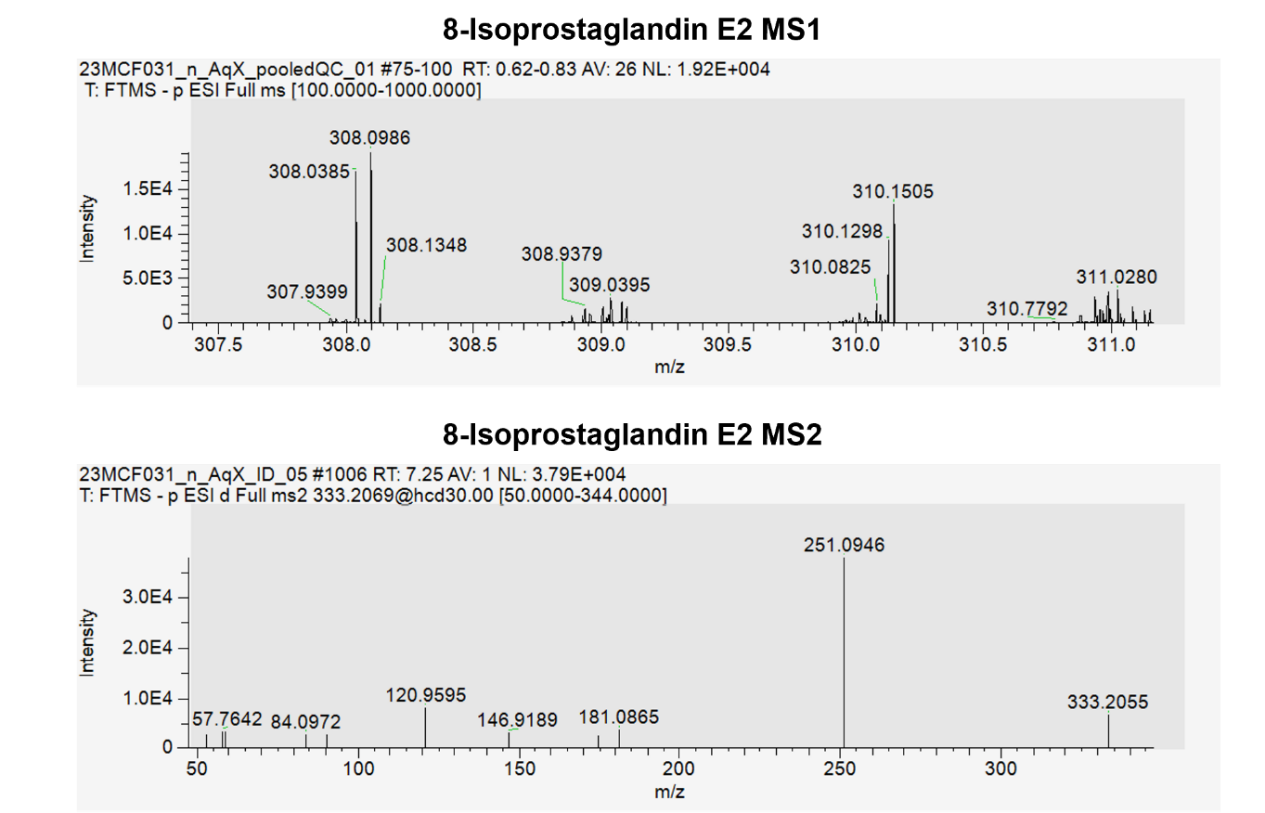


**Fig. S16.** Representative spectra for feature annotated as 8-isoprostaglandin E2 (level 4) in negative mode.


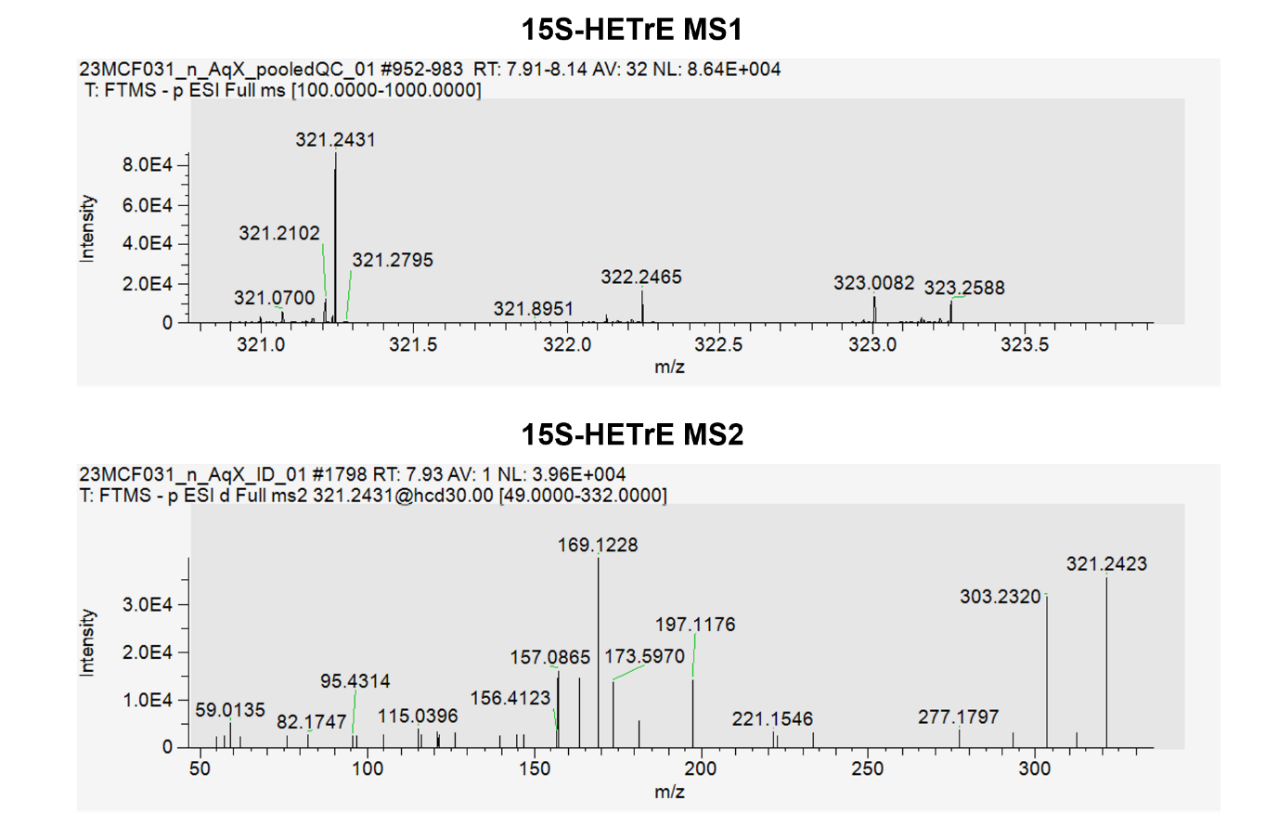


**Fig. S17.** Representative spectra for feature annotated as 15S-HETrE (level 3) in negative mode.


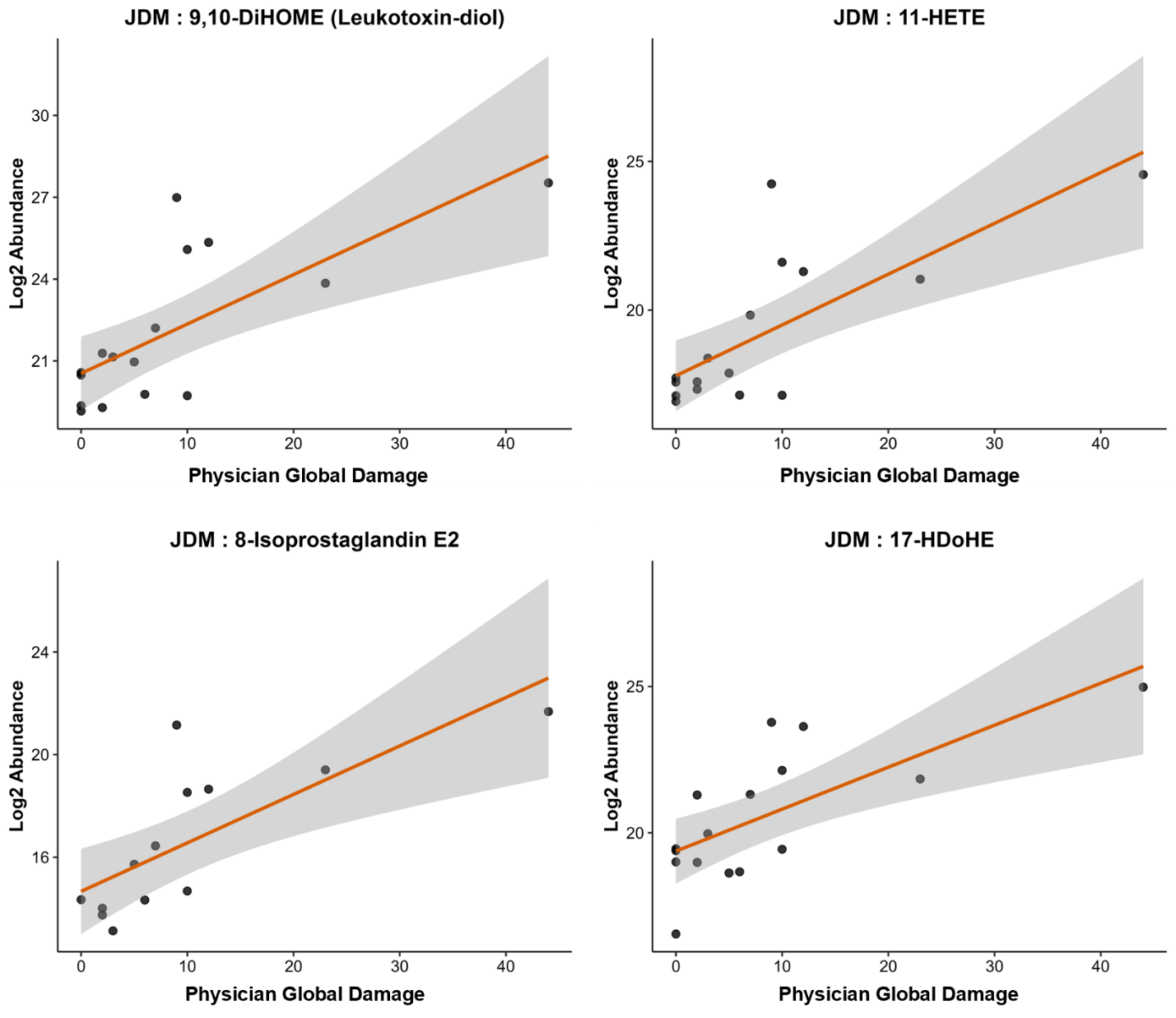


**Fig. S18.** Bioactive lipids with significant positive correlation with Physician Global Damage scores in addition to leukotriene B4.
